# Influence of a Polyphenol-Enriched Protein Powder on Exercise-Induced Inflammation and Oxidative Stress in Athletes: A Randomized Trial Using a Metabolomics Approach

**DOI:** 10.1371/journal.pone.0072215

**Published:** 2013-08-15

**Authors:** David C. Nieman, Nicholas D. Gillitt, Amy M. Knab, R. Andrew Shanely, Kirk L. Pappan, Fuxia Jin, Mary Ann Lila

**Affiliations:** 1 Human Performance Laboratory, Appalachian State University, North Carolina Research Campus, Kannapolis, North Carolina, United States of America; 2 Dole Nutrition Research Laboratory, North Carolina Research Campus, Kannapolis, North Carolina, United States of America; 3 Metabolon, Inc., Durham, North Carolina, United States of America; 4 Plants for Human Health Institute, North Carolina State University, North Carolina Research Campus, Kannapolis, North Carolina, United States of America; University of Sao Paulo, Brazil

## Abstract

**Objectives:**

Polyphenol supplementation was tested as a countermeasure to inflammation and oxidative stress induced by 3-d intensified training.

**Methods:**

Water soluble polyphenols from blueberry and green tea extracts were captured onto a polyphenol soy protein complex (PSPC). Subjects were recruited, and included 38 long-distance runners ages 19–45 years who regularly competed in road races. Runners successfully completing orientation and baseline testing (N = 35) were randomized to 40 g/d PSPC (N = 17) (2,136 mg/d gallic acid equivalents) or placebo (N = 18) for 17 d using double-blinded methods and a parallel group design, with a 3-d running period inserted at day 14 (2.5 h/d, 70% VO_2max_). Blood samples were collected pre- and post-14 d supplementation, and immediately and 14 h after the third day of running in subjects completing all aspects of the study (N = 16 PSPC, N = 15 placebo), and analyzed using a metabolomics platform with GC-MS and LC-MS.

**Results:**

Metabolites characteristic of gut bacteria metabolism of polyphenols were increased with PSPC and 3 d running (e.g., hippurate, 4-hydroxyhippurate, 4-methylcatechol sulfate, 1.8-, 1.9-, 2.5-fold, respectively, P<0.05), an effect which persisted for 14-h post-exercise. Fatty acid oxidation and ketogenesis were induced by exercise in both groups, with more ketones at 14-h post-exercise in PSPC (3-hydroxybutyrate, 1.8-fold, P<0.05). Established biomarkers for inflammation (CRP, cytokines) and oxidative stress (protein carbonyls) did not differ between groups.

**Conclusions:**

PSPC supplementation over a 17-d period did not alter established biomarkers for inflammation and oxidative stress but was linked to an enhanced gut-derived phenolic signature and ketogenesis in runners during recovery from 3-d heavy exertion.

**Trial Registration:**

ClinicalTrials.gov, U.S. National Institutes of Health, identifier: NCT01775384

## Introduction

Polyphenols are a large class of colorful, plant-based, phenolic organic compounds including tannins, lignins, and flavonoids. Flavonoids, the major polyphenolic subgroup, comprise more than 6,000 compounds classified into six subgroups [1]. In vitro, animal, and epidemiologic studies support multiple flavonoid-related physiologic and health effects, including anti-oxidative, anti-inflammatory, immune-regulatory, anti-carcinogenic, and cardioprotective influences [2], [3].

Prolonged and intensive exercise induces transient immune dysfunction, inflammation, oxidative stress, muscle damage, and muscle soreness [4], [5]. Ibuprofen is a popular drug among runners to help cope with the physiologic demands of training and competition, but several recent studies question both its efficacy and safety [6], [7]. There is growing interest in the use of polyphenol-rich fruit/vegetable extracts to mitigate exercise-induced physiologic stress and function as ibuprofen substitutes [8]. For any particular plant extract studied within an exercise context, few papers are available, and research designs vary widely with regard to the supplementation dose (near normal daily intake to supramaximal volumes) and regimen (acute to multiple weeks and months), the type of exercise stress (moderate to prolonged and intensive), type of subject (trained and untrained), and outcome measures (predominantly oxidative stress) [9]–[21]. As a result, no clear consensus has arisen from the published literature regarding the efficacy of plant extracts in serving as countermeasures to exercise-induced physiologic stress.

Metabolomics is the simultaneous measurement of all detectable small molecules or metabolites present in biological samples such as biofluids, tissues, and cellular extracts to elucidate the effect of a particular stimulus on metabolic pathways [22]. The use of metabolomics in nutritional sciences is gaining momentum, and global metabolomics profiling was utilized in this study to help capture the potential influence of a polyphenol- and protein-rich supplement in countering physiologic stress indicators associated with an intensified 3-day exercise training period. Metabolomics is particularly useful in interpreting human responses to polyphenol manipulation of the diet, and improves the capacity to capture their complex and subtle influences on whole body metabolism and physiology.

We hypothesized that a high-dose polyphenol supplement would serve as a partial countermeasure to inflammation and oxidative stress induced by a 3-day intensified training period, and that metabolomics would capture this benefit more effectively than traditional biomarkers.

## Methods

The protocol for this trial and supporting CONSORT checklist are available as supporting information; see Checklist S1 and Protocol S1.

### Subjects and Research Design

**Figure 1:**
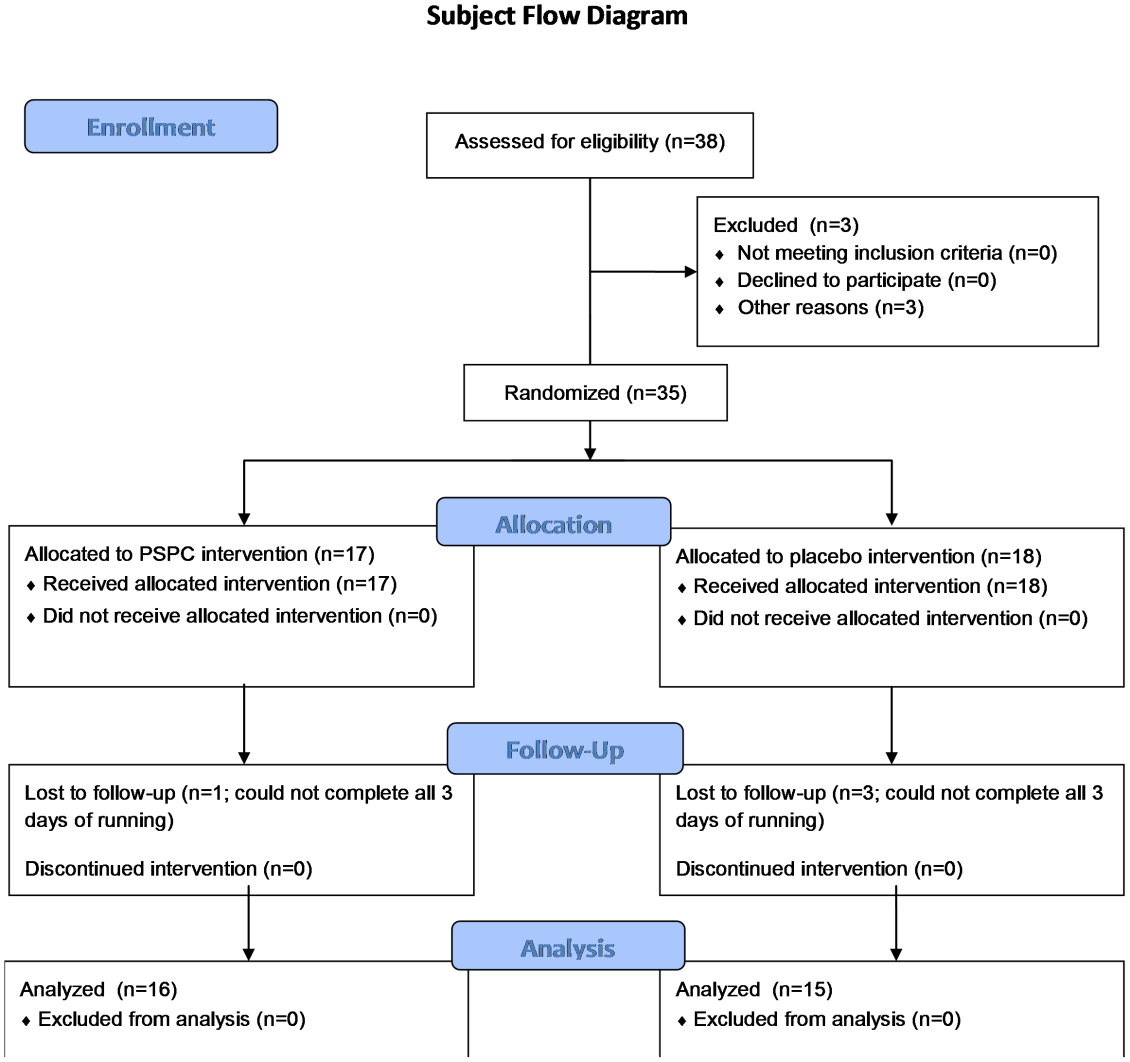
Subject flow diagram.

Subject recruitment was conducted via mass advertising to running clubs in the Charlotte, NC, metropolitan area. Subjects were recruited by the Human Performance Laboratory Research Manager, and included 38 healthy, non-smoking long distance male or female runners ages 19–45 years who regularly competed in marathon and half-marathon road races and were capable of running 2.5 h at high intensity on laboratory treadmills. During the study, subjects consented to train normally, maintain weight, and avoid the use of all herbs and medications known to affect inflammation and immune function for the duration of the study (in particular, all non-steroidal anti-inflammatory drugs). Subjects also agreed to avoid all vitamin and mineral supplements above 100% the U.S. Daily Value. Runners successfully completing orientation and baseline testing (N = 35) were randomized by the research manager using 1∶1 allocation and a random number table (without blocking) to intervention [polyphenol soy protein complex (PSPC)] (N = 17) or placebo groups (N = 18), with supplements administered over a 17-d period using double-blinded methods and a parallel group design. PSPC and placebo supplements were prepared by the Dole Nutrition Institute (NDG, FJ), with coding concealed until after all data were collected. All other investigators, study personnel, and subjects were blinded to the type of supplement used by the two groups during the study. All subjects signed informed consent forms, and all study procedures were approved by the Institutional Review Board at Appalachian State University (ASU). As summarized in Figure 1, data were analyzed from subjects completing all aspects of the study (N = 16 PSPC, N = 15 placebo). The study was conducted during the winter/spring of 2012 at the ASU Human Performance Laboratory at the North Carolina Research Campus in Kannapolis, NC.

### Baseline Testing

Two weeks prior to the 3-day period of exercise (2.5-h/day running bouts), subjects were tested for VO_2max_ during a graded, treadmill test with the Cosmed FitMate metabolic device (Cosmed, Rome, Italy). Body composition was measured with the Bod Pod body composition analyzer (Life Measurement, Concord, CA). Demographic and training histories were acquired with questionnaires. A blood sample was collected mid-afternoon to coincide with the same time of the day for the post-supplementation blood draw.

### Supplementation Product and Procedures

Gallic acid, caffeine and the flavan-3-ol standards: gallocatechin (GC); epigallocatechin (EGC); epicatechin (EC); catechin (C); epigallocatechin gallate (EGCG); gallocatechin gallate (GCG); catechin gallate (CG) and epicatechin gallate (ECG) were all obtained from Sigma Aldrich (St. Louis, MO). All other compounds and solvents were purchased from Fisher (Fair Lawn, NJ).

Green tea extract (GTE, product# Std +101) was purchased from Finlay Tea Solutions US Inc. (Florham Park, NJ). Liquid blueberry pomace water extract was a gift from Milne Fruit (Prosser, WA). Soy protein isolate (SPI) was obtained from Archer Daniels Midland (Ardex F, ADM, Decatur IL). All clinical trial study materials were provided by Nutrasorb LLC (North Brunswick, NJ), and supplements prepared by the Dole Nutrition Research Institute. Blueberry polyphenol-soy protein complex (22 kg) and green tea-polyphenol soy protein complex (8 kg) were produced individually then blended to obtain a 3∶1 blueberry-green tea-polyphenol soy protein complex (PSPC). Placebo was prepared from SPI, with non-polyphenolic food colorings (mixture of FD&C Blue #1 and FD&C Re #40) added to approximate the purple hue of PSPC.

Briefly, 4 kg of GTE was dissolved into 40 L of water heated to 45°C and SPI (4 kg) was added and mixed for 30 min until uniform. The mixture was vacuum-evaporated to remove excess water and then freeze-dried and ground to obtain the green tea-polyphenol and soy protein complex. To produce the blueberry polyphenol-soy protein complex, 44 L of blueberry pomace extract was mixed with 176 L of water (5× dilution) until uniform then SPI (100 g/L; 22 kg) was added to the diluted blueberry pomace extract and mixed. The mixture was centrifuged to separate solids from the supernatant. The blueberry solids were placed on trays and dried with a circulation bath set to 35°C under vacuum and the dried cake ground to a fine powder.

The concentration of total polyphenols sorbed per gram for each of the SPI’s was quantified by the Folin-Ciocalteu method [23] with samples read at 760 nm using a UV/Vis spectrophotometer (Beckman Coulter DU520) against a gallic acid standard curve. Values were obtained for both the green tea and blueberry SPI’s individually, as well as the matrix SPI (placebo) and PSPC (3∶1 blueberry-green tea, intervention).

Flavan-3-ol standards (0.1 mg/ml) were dissolved in 1∶1 acetonitrile:H_2_O, serially diluted and submitted for analysis. For blueberry anthocyanins chromatographic separation was accomplished with a Phenomenex Luna C18(2) (4.6×250 mm, 5 µm) reverse phase column (Torrance, CA) maintained at 30°C. Elution was at a 700 µl/min flow rate using mobile phase A (H_2_O with 0.1% formic acid) and mobile phase B (acetonitrile with 0.1% formic acid). LC gradient: (0–2 min, 5% B; 2–10 min, 5–20% B: 10–18 min, 20% B; 18–30 min, 20–95% B; 30–40 min, 95–5% B). Detection was via low-resolution electrospray mass spectrometry performed on a Thermo Scientific LTQ Velos ion-trap mass spectrometer fitted with an electrospray interface (ESI) operating in full scan MS mode from 50 to 1000 amu. MS/MS scan was performed using data dependent mode. Samples were analyzed using both negative and positive ionization modes. ESI-MS parameters were as follows: potential of ESI source, 4 kV; capillary temperature, 420°C; sheath gas, 58 arbitrary units; auxiliary gas, 20 arbitrary units. For green tea flavan-3-ols chromatographic separation was accomplished with a Phenomenex Kinetex C18 column (4.6×150 mm, 2.6 µm) reverse phase column (Torrance, CA) maintained at 30°C. Elution was at a 700 µl/min flow rate using mobile phase A (H_2_O with 0.1% formic acid) and mobile phase B (acetonitrile with 0.1% formic acid). LC gradient: (0–20 min, 2–70% B; 20–24 min, 70–100% B: 24–30 min, 2% B). Detection was via low-resolution electrospray mass spectrometry performed on a Thermo Scientific LTQ Velos ion-trap mass spectrometer fitted with an electrospray interface (ESI) operating in full scan MS mode from 100 to 1500 amu. MS/MS scan was performed using data dependent mode. Samples were analyzed using both negative and positive ionization modes. ESI-MS parameters were as follows: potential of ESI source, 4 kV; capillary temperature, 315°C; sheath gas, 45 arbitrary units; auxiliary gas, 10 arbitrary units.

The individual flavan-3-ols contained in the green tea polyphenol SPI complex were quantified by LC-MS/MS (LTQ Velos, Thermo Scientific) [24]. Quantitation was accomplished by comparison with calibration curves for all the authentic flavan-3-ols obtained and based on the following transitions: GC and EGC, MS^2^ 305.10>179.00 (305.10>219.00 and 305.10>261.00 as qualifiers); C and EC, MS^2^ 289.10>179.00 (289.10>205.00 and 289.10>245.00 as qualifiers); EGCG and GCG, MS^2^ 457.10>169.00 (457.10>305.00 and 457.10>331.00 as qualifiers); CG and ECG, MS^2^ 441.10>169.00 (441.10>271.00 and 441.10>289.00 as qualifiers); and internal standard, MS^2^ 292.10>181.00 (292.10>208.00 and 292.10>248.00 as qualifiers) at CID collision energy (CE of 35.0%).

The caffeine content of the green tea, blueberry, both SPI complexes, the matrix SPI and PSPC was analyzed with UHPLC (Agilent 1200) and DAD detection equipped with a Kinetex C18 column (100 mm×4.6 mm, 2.6 µm particle size, Phenomenex) at 20^○^C, a 700 µl/min flow rate, and 5 µl injection volume [24], [25]. LC gradient started at 98% A (0.1% formic acid in water) ­ 2% B (0.1% formic acid in acetonitrile) and ramped to 85%A-15% B at 8.00 min, held for 2.60 min and equilibrated for an additional 4.00 min to the starting condition. Quantitation was performed at 273 nm with reference wavelength at 360 nm.

Supplementation took place over a 17-day period, including a 14-day pre-exercise period, and then during each day of the 3-day intensified exercise period (see below). Subjects were randomized to PSPC or placebo (parallel group design, double-blinded treatments), and consumed 20 g mixed in 237 ml water in the morning, and then 20 g again at lunch (thus 40 g/day). Compliance to the supplementation regimen was checked through email messages, and during the post-supplementation lab visits.

### Exercise Sessions

Subjects trained normally during the 2-week supplementation, and then participated in a 3-day period of intensified exercise. In the morning of the first exercise session, subjects consumed the normal supplement portion (one tablespoon of PSPC or placebo in one cup of water) and breakfast (ad libitum). A standardized meal consisting of Boost Plus at 10 kcal/kg was ingested at 12∶00 noon. The normal lunch-time PSPC or placebo supplement was delayed until after the blood draw at 2∶30 pm. Subjects reported to the Human Performance Laboratory at 2∶30 pm and provided blood samples in a rested, seated position. Subjects next ingested the PSPC or placebo supplement dose. At 3∶00 pm, subjects ran on treadmills for 2.5-h at approximately 70% VO_2max_. Water was given ad libitum throughout the 2.5-h exercise bouts, with no other beverage or food allowed. Subjects ingested another 1 tablespoon dose of PSPC or placebo mixed in water after one hour of exercise. Heart rate, rating of perceived exertion (RPE), and distance run were recorded every 30 minutes during the bout, with oxygen consumption and ventilation measured after one hour of exercise. Subjects repeated this schedule for the next two days, but without pre-exercise blood draws. Blood samples were taken immediately following the third exercise bout on the third day, and 14-h post-exercise the following morning. A symptom log was administered during the final blood draw session. The symptom logs included questions on digestive health (constipation, heartburn, bloating, diarrhea, and nausea), hunger levels (morning, afternoon, and evening), energy levels (morning, afternoon, and evening), sickness (fever, cough, sore throat, stuffy nose, runny nose, and headache), pain (joint, muscle, and back), allergies, stress level, focus/concentration, and overall well-being. Subjects indicated responses using a 12- point Likert scale, with 1 relating to “none at all”, 6 “moderate”, and 12 “very high”.

### Comprehensive Diagnostics Chemistry Panel and Complete Blood Count

A serum chemistry panel, and complete blood counts (CBC) were performed by our clinical hematology laboratory. The CBC was conducted using a Coulter Ac.TTM 5Diff Hematology Analyzer (Beckman Coulter, Inc., Miami, FL). Shifts in plasma volume due to exercise were calculated using the equation of Dill and Costill [26].

### Plasma Cytokines

Total plasma concentrations of six inflammatory cytokines [tumor necrosis factor alpha (TNFα), granulocyte colony stimulating factor (GCSF), monocyte chemoattractant protein 1 (MCP), IL-6, IL-8, and IL-10] were determined using an electrochemiluminescence based solid-phase sandwich immunoassay (Meso Scale Discovery, Gaithersburg, MD, USA). All samples and provided standards were analyzed in duplicate, and the intra-assay CV ranged from 1.7 to 7.5% and the inter-assay CV 2.4 to 9.6% for all cytokines measured. Pre- and post-exercise samples for the cytokines were analyzed on the same assay plate to decrease inter-kit assay variability.

### Oxidative Stress and Antioxidant Capacity

Protein carbonyls were measured according to protocol (Cayman Chemical, 10005020). 220 µL of sample supernatant were pipetted in duplicate into a micro-well plate and read at 370 nm (Synergy H1 Hybrid Reader, BioTek Instruments Inc., Winooski, Vermont). Total protein was determined using the duplicate sample aliquot by adding 20 µL of sample to the micro-plate in duplicate followed by 180 µL of guanidine hydrochloride, and 200 µL of BSA standards and read at 280 and 260 nm. Plasma F_2_-isoprostanes were determined using gas chromatography mass spectrometry (GC-MS) [27]. Plasma was collected from heparinized blood, immediately flash-frozen in liquid nitrogen, and stored at −80°C. Immediately prior to assay plasma samples were thawed. The samples were used to extract free F_2_-isoprostanes with deuterated [2H4] prostaglandin F_2_α added as an “internal” standard. The mixture was then added to a C18 Sep Pak column, followed by silica solid phase extractions. F_2_-isoprostanes were converted to pentafluorobenzyl esters, subjected to thin layer chromatography, and converted to trimethylsilyl ether derivatives. Samples were analyzed by a negative ion chemical ionization GC-MS using an Agilent 6890N gas chromatography interfaced to an Agilent 5975B inert MSD mass spectrometer (Agilent Technologies, Inc. Santa Clara, CA). Total plasma antioxidant power was determined by the ferric reducing ability of plasma (FRAP) assay, a single electron transfer reaction as previously described by Benzie et al. [28].

### Metabolomics

The non-targeted metabolic profiling instrumentation employed for this analysis combined three independent platforms: ultrahigh performance liquid chromatography/tandem mass spectrometry (UHPLC/MS/MS^2^) optimized for basic species, UHPLC/MS/MS^2^ optimized for acidic species, and gas chromatography/mass spectrometry (GC/MS) [29], [30]. Blood samples were collected in serum separator tubes, allowed to stand at room temperature for 15–20 min, centrifuged at 2500 RPM for 10 minutes at 4°C, aliquoted, and then stored at −80°C until analysis. For each serum sample, 100 µL was used for analyses. Using an automated liquid handler (Hamilton LabStar, Salt Lake City, UT), protein was precipitated from the plasma with methanol that contained four standards to report on extraction efficiency. The resulting supernatant was split into equal aliquots for analysis on the three platforms. Aliquots, dried under nitrogen and vacuum-desiccated, were subsequently either reconstituted in 50 µL 0.1% formic acid in water (acidic conditions) or in 50 µL 6.5 mM ammonium bicarbonate in water, pH 8 (basic conditions) for the two UHPLC/MS/MS^2^ analyses or derivatized to a final volume of 50 µL for GC/MS analysis using equal parts bistrimethyl-silyl-trifluoroacetamide and solvent mixture acetonitrile:dichloromethane:cyclohexane (5∶4:1) with 5% triethylamine at 60°C for 1 h. In addition, three types of controls were analyzed in concert with the experimental samples: aliquots of a well-characterized human plasma pool served as technical replicates throughout the data set, extracted water samples served as process blanks, and a cocktail of standards spiked into every analyzed sample allowed instrument performance monitoring. Experimental samples and controls were randomized across platform run days.

For UHLC/MS/MS^2^ analysis, aliquots were separated using a Waters Acquity UPLC (Waters, Millford, MA) and analyzed using an LTQ mass spectrometer (Thermo Fisher Scientific, Inc., Waltham, MA) which consisted of an electrospray ionization (ESI) source and linear ion-trap (LIT) mass analyzer. The MS instrument scanned 99–1000 m/z and alternated between MS and MS^2^ scans using dynamic exclusion with approximately 6 scans per second. Derivatized samples for GC/MS were separated on a 5% phenyldimethyl silicone column with helium as the carrier gas and a temperature ramp from 60°C to 340°C and then analyzed on a Thermo-Finnigan Trace DSQ MS (Thermo Fisher Scientific, Inc.) operated at unit mass resolving power with electron impact ionization and a 50–750 atomic mass unit scan range. Metabolites were identified by automated comparison of the ion features in the experimental samples to a reference library of chemical standard entries that included retention time, molecular weight (m/z), preferred adducts, and in-source fragments as well as associated MS spectra, and were curated by visual inspection for quality control using software developed at Metabolon Inc. (Durham, NC) [31].

### Statistical Analysis

The primary outcome measures for this study were the metabolomics data, with all other inflammation and oxidative stress biomarkers regarded as secondary outcomes. All data are expressed as mean ± SD. Group data in Table 1 were compared using student t-tests. Our power analysis showed that at an effect size of 0.7 and alpha of 0.05, N = 30 in a randomized, parallel group design will provide a power of 0.90 for selected inflammation parameters (in particular, IL-6 and CRP). The biomarker data (Table 2) were analyzed using a 2 (condition) × 4 (time) repeated-measures ANOVA, between-subjects design. For the metabolomics statistical analyses and data display purposes, any missing values were assumed to be below the limits of detection and these values were imputed with the compound minimum (minimum value imputation). Statistical analysis of log-transformed data was performed using “R” (http://cran.r-project.org/), which is a freely available, open-source software package. Two-way ANOVA with post-hoc contrasts (t-tests) was performed to compare data between experimental groups. An estimate of the false discovery rate (Q-value) was calculated to take into account the multiple comparisons that normally occur in metabolomic-based studies, with Q<0.10 used as an indication of high confidence in a result [32]. Other lines of evidence were also taken into consideration when the Q-value exceeded 0.10, including the inclusion of a metabolite in a common pathway with a highly significant compound, or that the metabolite resided in a similar functional biochemical family with other significant compounds.

**Table 1:** Subject characteristics (mean±SD).

VARIABLE	PSPC*(N = 16)	Placebo (N = 15)	P-Value
Gender	M = 11, F = 5	M = 7, F = 8	0.378
Age (years)	33.7±6.8	35.2±8.7	0.593
Height (m)	1.74±0.7	1.72±1.0	0.589
Body mass (kg)	70.9±2.8	70.1±3.2	0.848
Body composition (% fat)	18.4±6.6	19.7±7.8	0.605
Peak aerobic power (ml^.^kg^.−1^min^−1^)	57.2±7.2	54.3±1.6	0.232
Heart rate max (beat^.^min^−1^)	182±14.8	178±11.0	0.306

* PSPC = polyphenol soy protein complex.

**Table 2:** Selected inflammation and oxidative stress/capacity markers in PSPC (N = 16) and placebo (N = 15) groups (mean±SD).

Variable	Baseline	Diet (14-dSupplementation)	Workout (Post-3d-Exercise)	Recovery (14 hPost-Exercise)	P-Value*
WBC (10^9^/L)					
PSPC	6.35±2.23	6.08±1.91	10.7±3.43	5.95±2.07	0.928
Placebo	6.23±1.51	6.54±1.58	10.9±3.48	5.96±1.49	<0.001
CRP (mg/L)					
PSPC	0.83±0.62	0.64±0.54	3.70±2.59	3.39±2.54	0.385
Placebo	0.84±0.57	0.95±0.87	4.74±2.88	4.53±2.86	<0.001
IL-6 (pg/ml)					
PSPC	0.44±0.40	0.49±0.42	5.11±3.46	0.53±0.43	0.437
Placebo	0.36±0.18	0.40±0.22	4.22±2.15	0.95±1.66	<0.001
IL-8 (pg/ml)					
PSPC	1.87±0.71	1.97±0.85	3.82±1.50	1.72±0.59	0.166
Placebo	1.81±0.57	1.57±0.46	4.46±2.26	1.81±0.96	<0.001
MCP-1 (pg/ml)					
PSPC	171±54.1	181±58.7	278±102	192±83.3	0.963
Placebo	167±40.4	173±36.0	281±91.6	194±62.3	<0.001
MPO (pmol/L)					
PSPC	40.9±19.1	43.8±31.4	61.0±27.6	32.6±13.7	0.685
Placebo	38.9±9.01	58.5±52.8	70.4±38.5	33.9±6.74	<0.001
FRAP (µmol/L)					
PSPC	519±108	511±96.1	623±80.4	552±95.4	0.395
Placebo	434±83	437±87.1	517±80.5	450±71.9	<0.001
Protein carbonyls (nmol/mg)					
PSPC	1.11±0.47	1.43±0.70	1.70±0.95	1.54±0.53	0.467
Placebo	0.75±0.25	0.98±0.50	1.36±0.61	1.39±0.51	<0.001

* The first P-value for each variable is the group×time interaction effect, and the second is the time effect.

Abbreviations: PSPC = polyphenol soy protein complex; WBC = white blood cell count; CRP = C-reactive protein; IL = interleukin; MCP = monocyte chemoattractant protein; MPO = myeloperoxidase; FRAP = ferric reducing ability of plasma (expressed as ascorbic acid equivalents).

## Results

Supplement characterization showed that green tea and blueberry SPI complexes and the uncomplexed SPI contained 90.0±0.6, 44.9±0.4 and 1.50±0.04 mg/g GAE of total phenolics, respectively. PSPC contained 53.4±1.3 mg/g GAE’s indicating adequate mixing of the blueberry and green tea SPI complexes in a 3∶1 ratio. The matrix SPI (placebo, including food coloring) contained 1.38±0.02 mg/g GAE’s indicating a low total phenolic content. Overall, the effective daily dose of PSPC (40 g) corresponded to 2,136 mg GAE’s. Both the total polyphenolic levels and individual catechin contents remained unchanged after 12 months storage at 5°C in the dark indicating a stable protein polyphenolic matrix. The enhanced stability of the polyphenolics sorbed to the maxtrix in PSPC is due to the protection afforded by the soy protein carrier [33].

Our LC-MS/MS data confirmed the presence of the flavan-3-ols in the green tea SPI complex as well as several hydroxycinnamic acids, glycosides of the flavonols quercetin and kaempferol and a single myricetin glycoside (See Figure S1). LC-MS/MS data also confirmed the presence of the numerous glycosides of the anthocyanins in the blueberry SPI complex. These components were consistent with that published for fresh blueberries [34] and consisted of the various glycosides of delphinidin, cyanidin, petunidin and malvidin (Figure S2). Several pentose conjugates were observed and MS data shows they are consistent with arabinose glycosides in blueberry (Tables S1 and S2).

The overall flavan-3-ol content of the green tea SPI complex was measured as 100.1 mg/g, meaning subjects received approximately 1001 mg of green tea flavan-3-ols in each 40 g daily dose of PSPC (3∶1 ratio of blueberry-green tea SPI complexes). Of the 8 flavan-3-ols detected (Figure S1), three, epigallocatechin gallate (EGCG), epigallocatechin (EGC), and epicatechin (EC), accounted for a little over 79% of this total. The caffeine content of the green tea SPI complex was determined to be 14.2±0.3 mg/g. No caffeine was found in the blueberry SPI complex or the matrix SPI. The caffeine content of PSPC was determined to be 3.44±0.02 mg/g and is consistent with a well mixed 3∶1 blueberry-green tea SPI complex mix.

Subjects reported 100% compliance to the supplementation regimen. Subject demographic and metabolic characteristics did not differ between PSPC and placebo groups, as summarized in Table 1. Additionally, no group differences in metabolic measures were found for the 2.5-h exercise periods over the 3-day intensified exercise period. For PSPC and placebo groups, 3-d averages during 7.5 h running for heart rate (HR) were 152±15.3 (82.1±3.6% HR_max_) and 151±9.7 bpm (84.2±3.6% HR_max_), respectively, (P = 0.930), oxygen consumption (VO_2_) were 37.8±3.9 (66.5±7.2% VO_2max_) and 36.5±3.2 ml^.^kg^−1.^min^−1^ (67.8±7.9% VO_2max_), respectively (P = 0.326), rating of perceived exertion (RPE) were 13.5±1.3 and 13.0±0.8, respectively (P = 0.300), distances run were 25.3±2.9 and 25.0±2.1 km/day, respectively (P = 0.777), weight changes were −1.8±0.9 and −1.5±0.7 kg/day, respectively (P = 0.327), and plasma volume shifts were −2.00±0.08% and −2.00±0.07%, respectively (P = 0.982). The intensity of effort during the 3-day exercise period did not differ between genders (data not shown), and gender×time interaction effects were non-significant for selected variables including protein carbonyls (P = 0.683), IL-6 (P = 0.804), and hippurate (P = 0.381). Thus for all outcome measures described in this report, genders have been combined within each group. Symptom logs showed no group differences for data collected at the end of 17-d supplementation (data not shown). Groups did not differ at any time point for measures included in the chemistry panel (all interaction effects, P>0.05) despite strong exercise induced increases for many of these including blood urea nitrogen (BUN), bilirubin, creatinine, alkaline phosphatase (ALK), and aspartate aminotransferase (AST) (time effects, P<0.001, data not shown).

**Figure 2:**
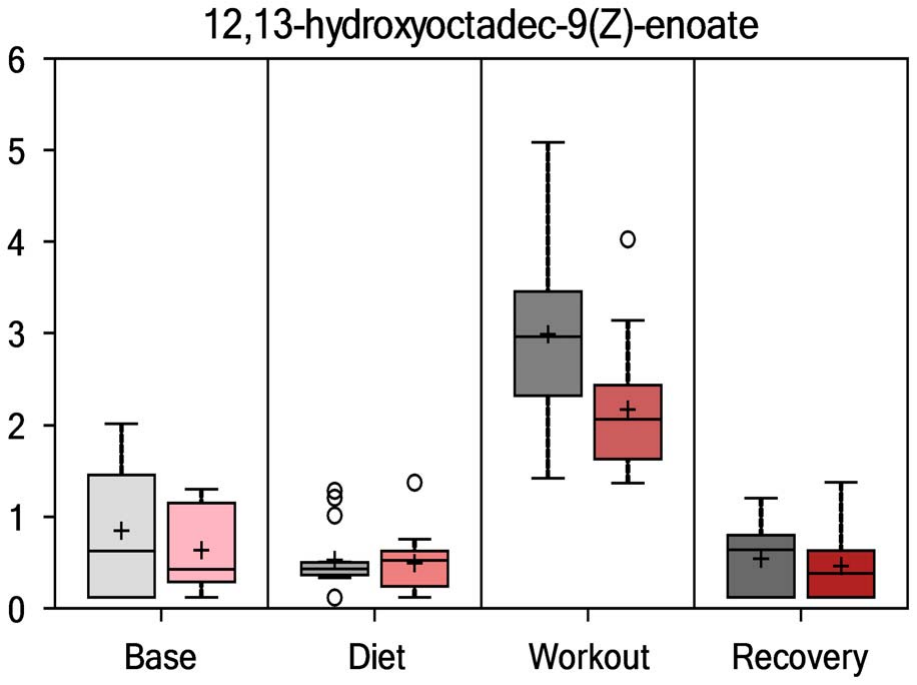
Serum 12,13-hydroxyoctadec-9(Z)-enoate (DHOME) (time effect, P<0.001; interaction effect, P = 0.9272). For all figures: PSPC = red bars; placebo = gray;+ = mean; ^____^ = median; ○ = extreme data points; box = upper and lower quartiles; whiskers = maximum and minimum of distribution; * = group contrast difference P<0.05. The vertical axis represents the median scaled intensity.

**Figure 3:**
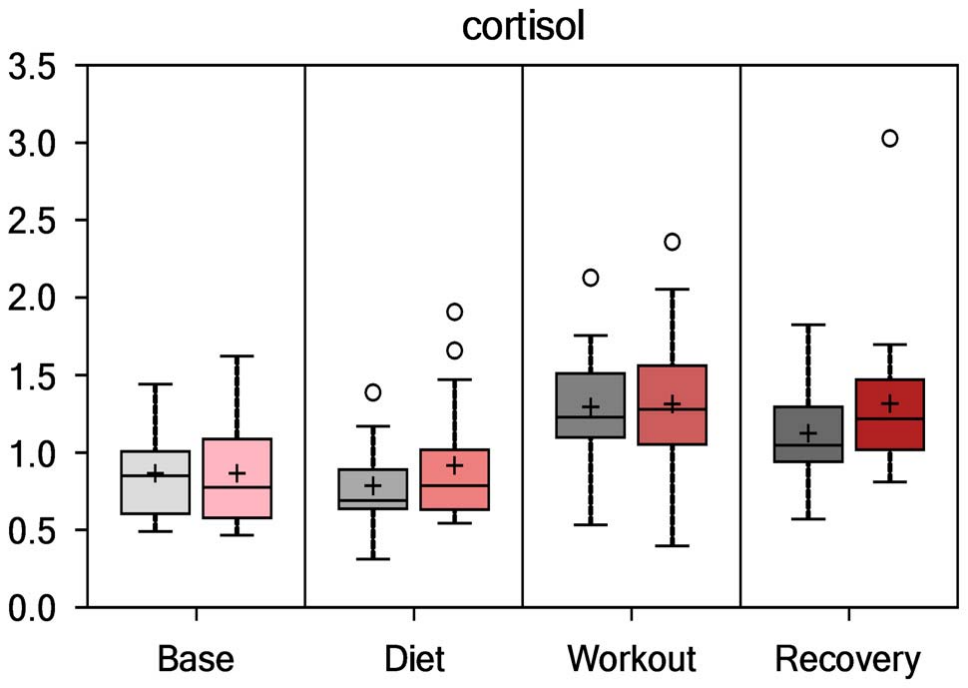
Serum cortisol (time effect, P<0.001; interaction effect, P = 0.466).

Data for selected inflammation and oxidative stress/capacity biomarkers are summarized for PSPC and placebo groups in Table 2. Groups did not differ pre-study for any of the variables listed in Table 2. The 3-day exercise period was associated with significant increases in both inflammation (white blood cell count, serum C-reactive protein, plasma IL-6, plasma monocyte chemoattractant protein-1, and plasma myeloperoxidase) and oxidative stress (plasma protein carbonyls), with no group differences in the pattern of change over time for these biomarkers. Figure 2 summarizes fold change data from the MS analysis for the serum oxidative stress indicator 12,13-hydroxyoctadec-9(Z)-enoate (DHOME). The vertical axis for all metabolomics variables represents the median scaled intensity for each metabolite. Both groups experienced strong fold increases in DHOME immediately post-exercise (time effect, P<0.001), but no differences in the overall pattern of change over time (P = 0.9272). Plasma F_2_-isoprostanes also increased in both groups following the 3-day exercise period, with no differences in the overall pattern of change over time (P = 0.583, data not shown). Figure 3 shows that serum cortisol (MS data, fold changes) increased significantly in both groups (time effect, P<0.001; interaction effect, P = 0.466), with an average fold increase of 1.54 immediately post-exercise for all subjects, and 1.44 the next morning (each time point, P<0.001). Plasma IL-10, tumor necrosis factor alpha (TNFα), and granulocyte colony stimulating factor (GCSF) also increased following 3-days exercise (P<0.001, data not shown), with no significant group differences in the pattern of change. The ferric reducing ability of plasma increased with exercise (P<0.001), with no difference in the pattern of change over time (P = 0.395).

The metabolomics analysis revealed 377 detectable compounds of known identity. Following log transformation and imputation with minimum observed values for each compound, repeated measures ANOVA identified significant time effects for 324 metabolites (primarily induced by the 3-day exercise period) and significant group × time interaction effects for 40 metabolites.

Time effects for both groups were notable for strong post-exercise increases in metabolites related to fatty acid oxidation including free fatty acids, acylcarnitines, 3-hydroxy-fatty acids, and dicarboxylic acids (data not shown). Other super pathways heavily affected by the 3-day exercise period included amino acid and carbohydrate metabolism, energy production, nucleotides, and cofactors and vitamins (data not shown). PSPC versus placebo supplementation had no effect on exercise-induced changes in these super pathways.

**Figure 4:**
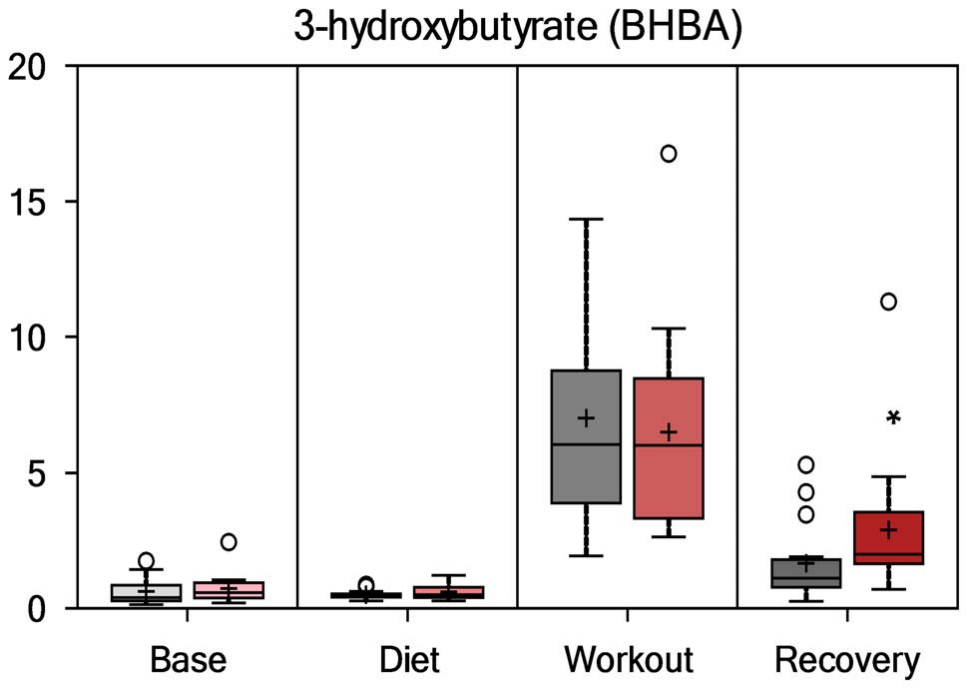
Serum 3-hydroxybutyrate (group contrast at 14-h recovery, P = 0.005, Q = 0.246).

**Figure 5:**
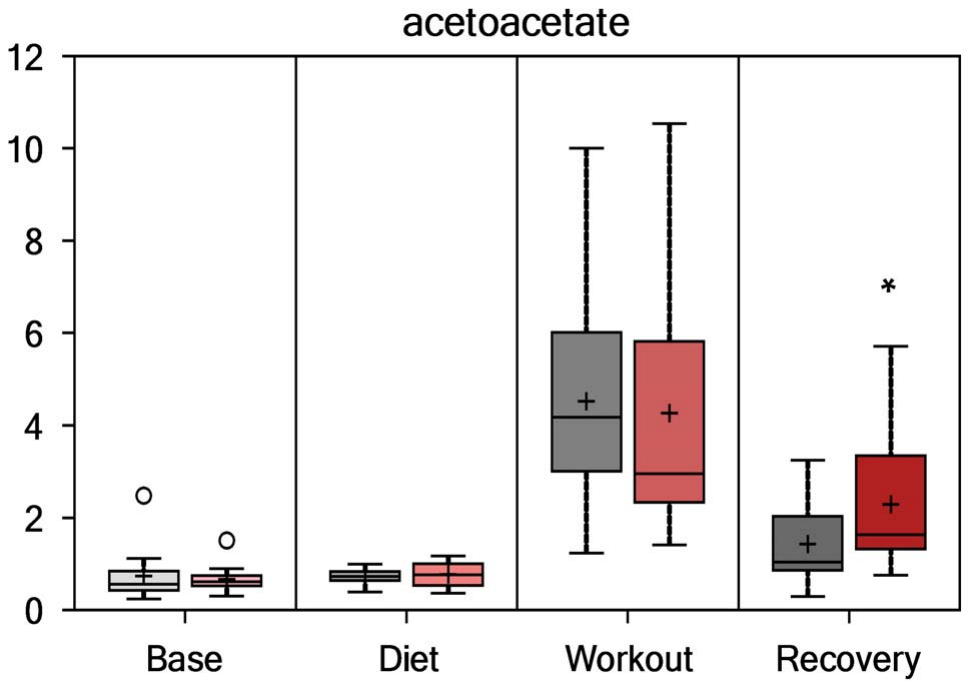
Serum acetoacetate (group contrast at 14-h recovery, P = 0.0105, Q = 0.265).

As summarized in Figures 4 and 5, 3-hydroxybutyrate (3-HBA) and acetoacetate (AcAc) were elevated in PSPC versus placebo at the recovery time point (fold differences, 1.75 and 1.60, respectively, both contrasts, P<0.01, with Q-values of 0.265 and 0.246, respectively), indicative of increased fatty acid oxidation and ketone synthesis. Group values for these metabolites did not differ at the other time points, and overall interaction effects were not significant (P = 0.1406 and 0.1035, respectively).

**Figure 6:**
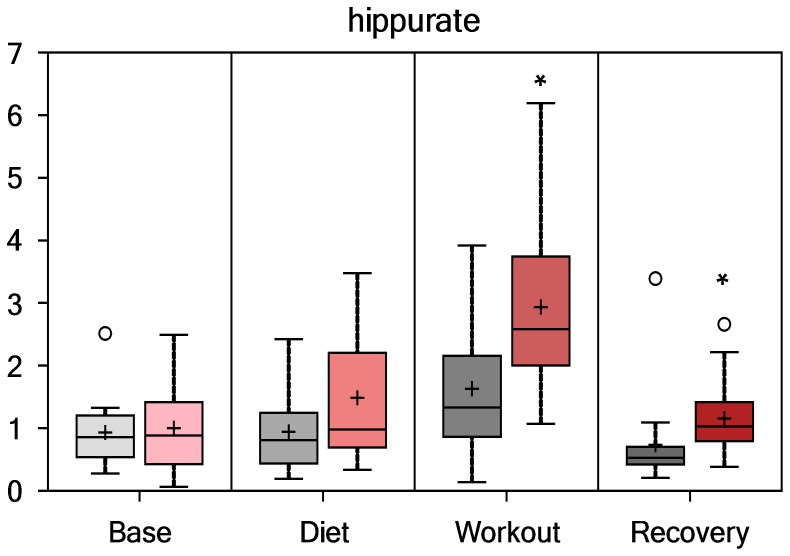
Serum hippurate (interaction effect, P = 0.0245; group contrasts immediately- and 14-h-post-exercise, P = 0.006 and 0.022, respectively; Q = 0.116 and 0.2648, respectively).

**Figure 7:**
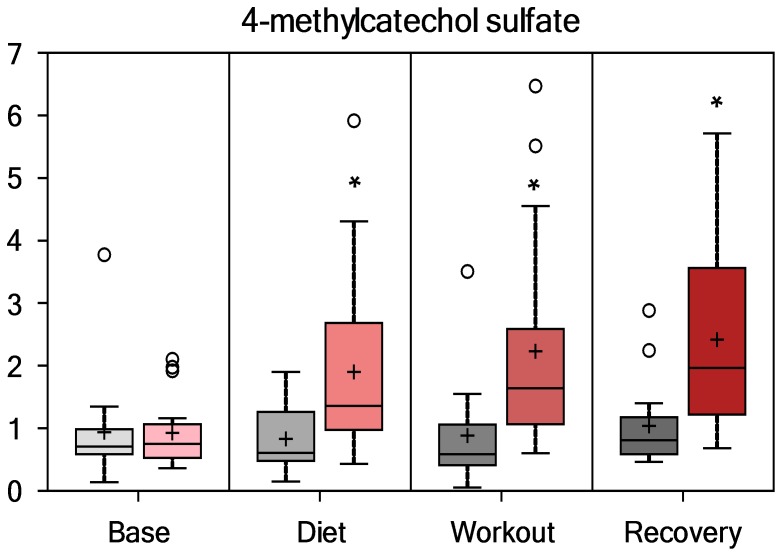
Serum 4-methylcatechol sulfate (interaction effect, P = 0.0017; group contrasts 14-days supplementation, P = 0.002, and immediately- and 14-h-post-exercise, P<0.001 and 0.0015, respectively; Q = 0.4392, 0.0083, and 0.1797, respectively).

**Figure 8:**
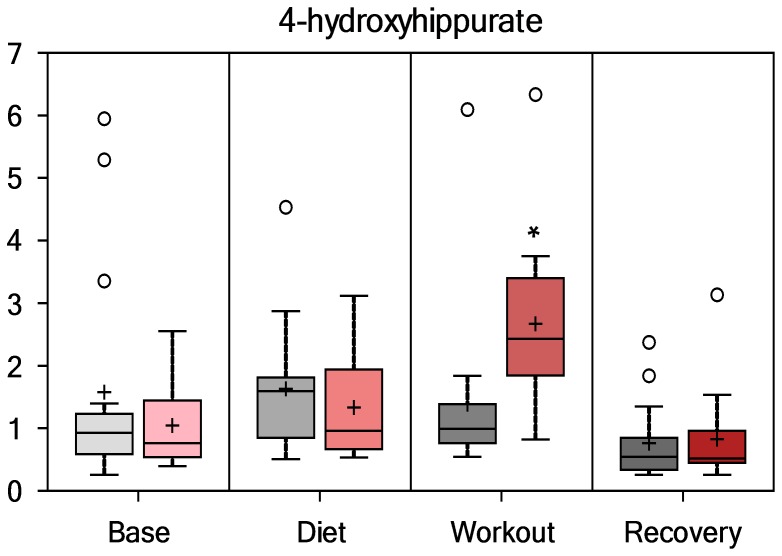
Serum 4-hydroxyhippurate (interaction effect, P = 0.0014; group contrast immediately-post-exercise, P = 0.0014).

**Figure 9:**
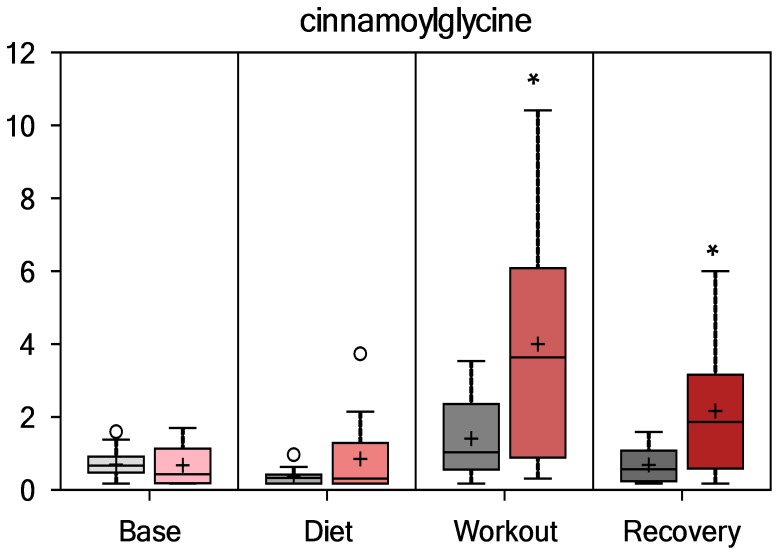
Serum cinnamoylglycine (interaction effect, P = 0.0017; group contrasts immediately- and 14-h-post-exercise, P = 0.008 and 0.009, respectively; Q = 0.1421 and 0.265, respectively).

Compounds with an aromatic group (i.e., benzoate sub-pathway) derived from colon bacterial degradation of blueberry and green tea extract phenolic compounds were elevated post-exercise in PSPC compared to placebo. As depicted in Figures 6, 7, 8, and 9, hippurate, 4-methylcatechol sulfate, 4-hydroxyhippurate, and cinnamoylglycine were elevated in PSPC, especially immediately following the 3-day period of exercise (interaction effects, P = 0.0245, 0.0017, 0.0014, and 0.0017, respectively). Other gut-derived metabolites from PSPC polyphenolics that were elevated post-exercise compared to placebo included 2-hydroxyhippurate (interaction effect, P = 0.0221), 3-hydroxyhippurate (P = 0.0352), catechol sulfate (P = 0.0687), and O-methylcatecholsulfate (P = 0.085). The pronounced increase in these gut-derived phenolics immediately post-exercise suggests a transient, exercise-induced increase in gut permeability.

**Figure 10:**
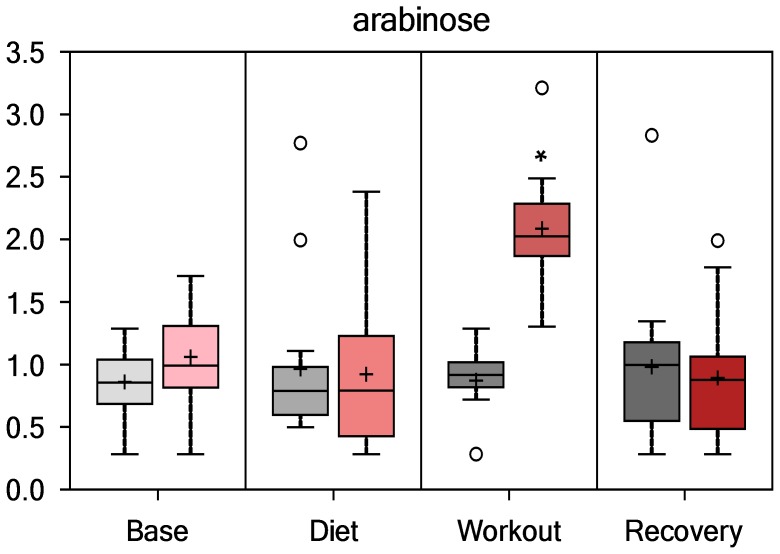
Serum arabinose (interaction effect, P<0.001; group contrast immediately-post-exercise, P<0.001, Q<0.001).

**Figure 11:**
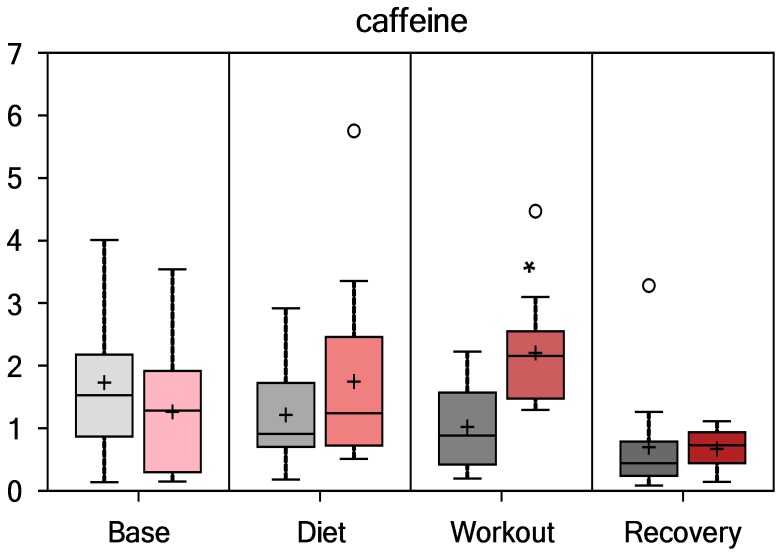
Serum caffeine (interaction effect, P<0.001; group contrast immediately-post-exercise, P = 0.0012, Q = 0.0626).

PSPC plant derived constituents showing significant interaction effects relative to placebo are shown in Figures 10 and 11, and included arabinose (P<0.001) and caffeine (P<0.001). Other xanthine metabolites with significant interaction effects (all, P<0.001) included paraxanthine, theophylline, 1-methylurate, and 1,7-dimethylurate. PSPC and placebo contrasts for these PSPC plant components were especially apparent immediately post-exercise.

## Discussion

The PSPC supplement used in this study contained a high dose of total phenolic compounds (2,136 mg GAE’s), the profiles of which were consistent with what is observed for green tea and fresh blueberries. The polyphenolic content was unchanged over the course of the study and in real terms is equivalent to the daily combined consumption of 3 whole cups of fresh blueberries (148 g/cup) and 1–1/3 cups of brewed green tea with its associated caffeine content (assuming 8 fl oz or 247 mls per cup).

The 3-day period of intensified exercise caused significant physiologic stress, inflammation, and oxidative stress in experienced long distance runners. Ingestion of the polyphenol-rich supplement compared to placebo over a 17-day period was not associated with any discernible countermeasure effects during recovery from the 3-day exercise period when using established biomarkers. The polyphenol-rich supplement was linked to a significant gut-derived phenolic signature that appeared to be more pronounced through exercise-induced increases in gut permeability, and an increase in fat oxidation and ketogenesis 14-h post-exercise.

The gut-derived phenolic signature was not associated with any positive effects using study outcome measures, but may provide some benefit to runners during stressful training, as inferred by other investigations. Many flavonoids possess strong anti-inflammatory, antioxidant, and other properties when studied in vitro using large doses of the purified form. The biological relevance of in vitro studies, however, is low because flavonoids are poorly absorbed in the human intestine, undergo active efflux, and are extensively conjugated and metabolically transformed, all of which diminish their bioactive capacities [35].

A large proportion of ingested plant polyphenols reach the colon, and there is a growing realization that the metabolites created from colonic bacterial degradation can be reabsorbed and exert bioactive effects [36]–[38]. Microbial phenolic metabolites can be eventually excreted in the urine, representing the largest proportion of polyphenol intake. As the gut-derived polyphenolic metabolites are taken up into the systemic circulation, they become a part of the “food metabolome” that interacts with the endogenous metabolome of the individual [36].

Microbial polyphenol metabolism produces a relatively small number of metabolites from the extremely diverse population of dietary polyphenols arriving in the colon, and these include simple phenols and derivatives of benzoic acid, phenylacetic acid, mandelic acid, phenylpropionic acid, and cinnamic acid [39]. The bacterial enzymes transform the polyphenols into smaller metabolites through deglycosylation, dehydroxylation, and demethylation [40]. This study showed that subjects consuming the PSPC product rich in anthocyanins and catechins exhibited an increase in simple phenols and benzoic acid derivatives such as hippurate and 4-methylcatechol sulfate. Other gut-derived phenolics that were expected from consumption of the blueberry and green tea supplement such as gallic acid, coumaric acid, protocatechuic acid, and quinic acid did not reach detectable levels [39]. However, most of these are commonly detected in matrices other than the serum samples used in this study such as urine and feces, or are in conjugated forms for which standards are lacking (Metabolon, personal communication). Mulder et al. [41] have previously shown that green tea ingestion results in a major excretion of hippuric acid into urine following microbial degradation. The colonic bacterial transformation of food polyphenols varies widely depending on the unique gut microbiota composition of the individual as influenced by genotype, diet, lifestyle, and other factors [42], and this variance was represented in our subjects as depicted in Figure 4.

Metabolomics is ideally suited as a methodology to investigate the shifts in gut-derived metabolites following polyphenol supplementation, and human trials are revealing an increasing number of metabolites that appear at high levels in the colon and systemic circulation [22]. The biological relevance for most of these gut-derived metabolites is still being explored, however [36], [42]–[47]. The microbial metabolites of dietary polyphenols have lower antioxidant and anti-inflammatory activities than do their parent compounds [41], but this may be offset by greater bioavailability for these smaller molecules. A unique contribution from our study was that the 3-day exercise period enhanced the gut-derived phenolic signature from the PSPC supplement through increased gastrointestinal permeability [48], and this may have positive implications for long-term bioactive influences. A growing number of studies indicate gut-derived polyphenol metabolites demonstrate both in vitro and in vivo anti-inflammatory and anti-oxidant influences [42]–[44], [47].

The athletes exhibited significant inflammation, oxidative stress, and muscle soreness after running at high intensity for 7.5 hours during the 3-day running period, and no apparent benefit was derived from the elevated gut-derived phenolic signature. Some studies that have followed athletes for a longer period of time following stressful exercise than used in this study report diminished oxidative stress, muscle damage and soreness, and a quicker recovery when consuming polyphenol-rich supplements [12], [18], [20]. McAnulty et al. [19], for example, showed that ingestion of 250 g/day blueberries for 6 weeks prior to 2.5 h of intensive running attenuated exercise-induced increases in F_2_-isoprostanes. Another consideration for future research is to focus on tissue-specific measures that may better capture subtle but significant phenolic-based anti-inflammatory and anti-oxidative influences. Little is known as to whether or not polyphenol-rich supplements have comparable effects in different types of athletic groups.

The daily polyphenol dose used in this study was equivalent to 2,136 mg/d gallic acid equivalents. This is substantially above the typical adult intake [49], but larger doses for a longer period of time may prove to be more efficacious. Subjects in the PSPC group showed an elevation in fat oxidation and ketogenesis 14-h post-exercise, an effect reported by other investigators [50], and which may become important with larger doses. Other types of outcome measures should be considered including acute respiratory illness. One study showed that 5-weeks intake of beer polyphenols significantly reduced the incidence of acute respiratory illness following a marathon competition [14].

PSPC intake was linked to some direct increases in plant constituents such as arabinose and caffeine. Arabinose is a pentose sugar that is linked to blueberry anthocyanins, and the strong increase post-exercise was probably caused by an interaction between colonic bacteria degradation of blueberry anthocyanins and an increase in gut permeability caused by the heavy exertion [48]. Green tea extract contains caffeine with a relatively short half-life, and the post-exercise increases probably reflects acute ingestion of the supplement product just before and one hour into the running bouts. The PSPC supplement caffeine content is reduced, since only 20% is sorbed to the protein surfaces during formulation. The increase in fat oxidation and ketogenesis 14-h post-exercise was more than likely related to PSPC supplement catechin-caffeine synergism [50].

In summary, this metabolomics-based investigation showed that 17 days of supplementation with a blueberry and green tea polyphenol-rich soy protein-based product (PSPC) caused a distinct gut-derived phenolic signature in long distance runners following a 3-day period of intensified running. Established measures of inflammation and oxidative stress did not indicate any discernible attenuation of exercise-induced changes in PSPC compared to placebo groups. The potential benefits of the gut-derived phenolic signature for the PSPC-supplemented athletic group require additional investigation, perhaps at the tissue level. Future research is also warranted to determine if longer-term polyphenol enrichment of the athletic diet mitigates the physiologic stress of heavy exertion, improves the speed of recovery, produces other benefits such as lowered incidence of acute respiratory illnesses, and has comparable effects in a variety of athletic groups.

## Supporting Information

Figure S1 **Negative ion mode total ion chromatogram (TIC) of green tea SPI (top), insert is expanded region (2–12 min).** Identified flavan-3-ols are: gallocatechin (1), epigallocatechin (2), catechin (3), epicatechin (4), epigallocatechin gallate (5), gallocatechin gallate (6), catechin gallate (7) and epicatechin gallate (8).
(TIF)

Figure S2 **Positive ion mode total ion chromatogram (TIC) of bluebery SPI (top), insert is expanded region (10–18 min).** Identified compounds by rentention time are: 11.99 - delphinidin 3-galactoside (Del-gal); 12.28 - delphinidin 3-glucoside (Del-glu); 12.69 - delphinidin pentose conjugate (Del-pent); 13.12 - petunidin 3-galactoside (Pet-gal); 13.41 - petunidin 3-glucoside (Pet-glu); 13.86 - petunidin pentose conjugate (Pet-pent); 14.14 - malvidin 3-galactoside (Mal-gal); 14.45 - malvidin 3-glucoside (Mal-glu); 14.93 - malvidin pentose conjugate 1 (Mal-pent1); 15.76 - malvidin pentose conjugate 2 (Mal-pent2) and 16.96 - malvidin acetoyl hexose conjugate (Mal-AcHex).
(TIF)

Table S1 **Mass spectral data for polyphenolic compounds identified in acidified methanol extract of blueberry SPI.**
(DOCX)

Table S2 **Mass spectral data for polyphenolic compounds identified in acidified methanol extract of green tea SPI.**
(DOCX)

Checklist S1 **CONSORT checklist.**
(DOC)

Protocol S1 **Human research protection application and protocol summary.**
(DOC)

## References

[1] U.S. Department of Agriculture, Agricultural Research Service (2011) USDA Database for the Flavonoid Content of Selected Foods, Release 3.0. Nutrient Data Laboratory Home Page: http://www.ars.usda.gov/nutrientdata/flav (accessed January 4, 2013).

[2] Galleano M, Calabro V, Prince PD, Litterio MC, Piotrkowski B, et al (2012) Flavonoids and metabolic syndrome. Ann N Y Acad Sci 1259: 87–94. 22758640 10.1111/j.1749-6632.2012.06511.x

[3] Landete JM (2012) Updated knowledge about polyphenols: functions, bioavailability, metabolism, and health. Crit Rev Food Sci Nutr 52: 936–948. 22747081 10.1080/10408398.2010.513779

[4] Nieman DC (2009) Immune function responses to ultramarathon race competition. Med Sportiva 13: 189–196.

[5] Powers SK, Jackson MJ (2008) Exercise-induced oxidative stress: cellular mechanisms and impact on muscle force production. Physiol Rev 88: 1243–1276. 18923182 10.1152/physrev.00031.2007 PMC2909187

[6] Nieman DC, Henson DA, Dumke CL, Oley K, McAnulty SR, et al (2006) Ibuprofen use, endotoxemia, inflammation, and plasma cytokines during ultramarathon competition. Brain Behav Immun 20: 578–584. 16554145 10.1016/j.bbi.2006.02.001

[7] Van Wijck K, Lenaerts K, Van Bijnen AA, Boonen B, Van Loon LJ, et al (2012) Aggravation of exercise-induced intestinal injury by ibuprofen in athletes. Med Sci Sports Exerc 44: 2257–2262. 22776871 10.1249/MSS.0b013e318265dd3d

[8] Nieman DC, Stear SJ, Castell LM, Burke LM (2010) A-Z of nutritional supplements: dietary supplements, sports nutrition foods and ergogenic aids for health and performance: part 15. Br J Sports Med 44: 1202–1205. 21156769 10.1136/bjsm.2010.078618

[9] Lyall KA, Hurst SM, Cooney J, Jensen D, Lo K, et al (2009) Short-term blackcurrant extract consumption modulates exercise-induced oxidative stress and lipopolysaccharide-stimulated inflammatory responses. Am J Physiol Regul Integr Comp Physiol 297: R70–R81. 19403859 10.1152/ajpregu.90740.2008

[10] Hurst RD, Wells RW, Hurst SM, McGhie TK, Cooney JM, et al (2010) Blueberry fruit polyphenolics suppress oxidative stress-induced skeletal muscle cell damage in vitro. Mol Nutr Food Res 54: 353–363. 19885847 10.1002/mnfr.200900094

[11] Panza VS, Wazlawik E, Ricardo Schütz G, Comin L, Hecht KC, et al (2008) Consumption of green tea favorably affects oxidative stress markers in weight-trained men. Nutrition 24: 433–442. 18337059 10.1016/j.nut.2008.01.009

[12] Trombold JR, Barnes JN, Critchley L, Coyle EF (2010) Ellagitannin consumption improves strength recovery 2–3 d after eccentric exercise. Med Sci Sports Exerc 42: 493–498. 19952825 10.1249/MSS.0b013e3181b64edd

[13] Nieman DC, Henson DA, Maxwell KR, Williams AS, McAnulty SR, et al (2009) Effects of quercetin and EGCG on mitochondrial biogenesis and immunity. Med Sci Sports Exerc 41: 1467–1475. 19516153 10.1249/MSS.0b013e318199491f

[14] Scherr J, Nieman DC, Schuster T, Habermann J, Rank M, et al (2012) Nonalcoholic beer reduces inflammation and incidence of respiratory tract illness. Med Sci Sports Exerc 44: 18–26. 21659904 10.1249/MSS.0b013e3182250dda

[15] Eichenberger P, Mettler S, Arnold M, Colombani PC (2010) No effects of three-week consumption of a green tea extract on time trial performance in endurance trained men. Int J Vitam Nutr Res 80: 54–64. 20533245 10.1024/0300-9831/a000006

[16] Jówko E, Sacharuk J, Balasińska B, Ostaszewski P, Charmas M, et al (2011) Green tea extract supplementation gives protection against exercise-induced oxidative damage in healthy men. Nutr Res 31: 813–821. 22118751 10.1016/j.nutres.2011.09.020

[17] Allgrove J, Farrell E, Gleeson M, Williamson G, Cooper K (2011) Regular dark chocolate consumption’s reduction of oxidative stress and increase of free-fatty-acid mobilization in response to prolonged cycling. Int J Sport Nutr Exerc Metab 21: 113–123. 21558573 10.1123/ijsnem.21.2.113

[18] Bowtell JL, Sumners DP, Dyer A, Fox P, Mileva KN (2011) Montmorency cherry juice reduces muscle damage caused by intensive strength exercise. Med Sci Sports Exerc 43: 1544–1551. 21233776 10.1249/MSS.0b013e31820e5adc

[19] McAnulty LS, Nieman DC, Dumke CL, Shooter LA, Henson DA, et al (2011) Effect of blueberry ingestion on natural killer cell counts, oxidative stress, and inflammation prior to and after 2.5 h of running. Appl Physiol Nutr Metab 36: 976–984. 22111516 10.1139/h11-120

[20] McLeay Y, Barnes MJ, Mundel T, Hurst SM, Hurst RD, et al (2012) Effect of New Zealand blueberry consumption on recovery from eccentric exercise-induced muscle damage. J Inter Soc Sports Nutr 9: 9–19. 10.1186/1550-2783-9-19 PMC3583121 22564864

[21] Morillas-Ruiz JM, Villegas Garcia JA, Lopez FJ, Vidal-Guevara ML, Zafrilla P (2006) Effects of polyphenolic antioxidants on exercise-induced oxidative stress. Clin Nutr 25: 444–453. 16426710 10.1016/j.clnu.2005.11.007

[22] McGhie TK, Rowan DD (2012) Metabolomics for measuring phytochemicals, and assessing human and animal responses to phytochemicals, in food science. Mol Nutr Food Res56: 147–158. 10.1002/mnfr.201100545 22162287

[23] Singleton VL, Rossi JA (1965) Colorimetry of total phenolics with phosphomolybdic-phosphotungstic acid reagents. Am J Enol Vitic 16: 144–158.

[24] Wu C, Xu H, Heritier J, Andlauer W (2012) Determination of catechins and flavonol glycosides in Chinese tea varieties. Food Chem 132: 144–149. 26434273 10.1016/j.foodchem.2011.10.045

[25] Sun B, Ricardo-da-Silva JM, Spranger I (1998) Critical factors of vanillin assay for catechins and proanthocyanidins. J Agr Food Chem 46: 4267–4274.

[26] Dill DB, Costill DL (1974) Calculation of percentage changes in volumes of blood, plasma and red cells in dehydration. J Appl Physiol 37: 247–248. 4850854 10.1152/jappl.1974.37.2.247

[27] Liu W, Morrow JD, Yin H (2009) Quantification of F2-isoprostanes as a reliable index of oxidative stress in vivo using gas chromatography-mass spectrometry (GC-MS) method. Free Radic Biol Med 47: 1101–1107. 19647073 10.1016/j.freeradbiomed.2009.07.028 PMC2749920

[28] Benzie IF, Strain JJ (1996) The ferric reducing ability of plasma (FRAP) as a measure of ‘antioxidant power’: the FRAP assay. Anal Biochem 239: 70–76. 8660627 10.1006/abio.1996.0292

[29] Ohta T, Masutomi N, Tsutsui N, Sakairi T, Mitchell M, et al (2009) Untargeted metabolomic profiling as an evaluative tool of fenofibrate-induced toxicology in Fischer 344 male rats. Toxicol Pathol 37: 521–535. 19458390 10.1177/0192623309336152

[30] Evans AM, DeHaven CD, Barrett T, Mitchell M, Milgram E (2009) Integrated, nontargeted ultrahigh performance liquid chromatography/electrospray ionization tandem mass spectrometry platform for the identification and relative quantification of the small-molecule complement of biological systems. Anal Chem 81: 6656–6667. 19624122 10.1021/ac901536h

[31] DeHaven CD, Evans AM, Dai H, Lawton KA (2010) Organization of GC/MS and LC/MS metabolomics data into chemical libraries. J Cheminform 2(1): 9 doi: 10.1186/1758-2946-2-9 20955607 10.1186/1758-2946-2-9 PMC2984397

[32] Storey JD, Tibshirani R (2003) Statistical significance for genomewide studies. Proc Natl Acad Sci USA 100: 9440–9445. 12883005 10.1073/pnas.1530509100 PMC170937

[33] Roopchand DE, Kuhn P, Rojo LE, Lila MA, Raskin I (2013) Blueberry polyphenol-enriched soybean flour reduces hyperglycemia, body weight gain and serum cholesterol in mice. Pharmacol Res 68: 59–67. 23220243 10.1016/j.phrs.2012.11.008 PMC3833590

[34] Grace M, Ribnicky DM, Kuhn P, Poulev A, Logendra S, et al (2009) Hypoglycemic activity of a novel anthocyanin-rich formulation from lowbush blueberry, Vaccinium angustifolium Aiton. Phytomed 16: 406–415. 10.1016/j.phymed.2009.02.018 PMC2718544 19303751

[35] González-Gallego J, García-Mediavilla MV, Sánchez-Campos S, Tuñón MJ (2010) Fruit polyphenols, immunity and inflammation. Br J Nutr 104 (Suppl 3)S15–S27. 20955647 10.1017/S0007114510003910

[36] van Duynhoven J, Vaughan EE, Jacobs DM, Kemperman RA, van Velzen EJ, et al (2011) Metabolic fate of polyphenols in the human superorganism. Proc Natl Acad Sci U S A 15 108 (Suppl 1)4531–4538. 10.1073/pnas.1000098107 PMC3063601 20615997

[37] Roowi S, Stalmach A, Mullen W, Lean ME, Edwards CA, et al (2010) Green tea flavan-3-ols: colonic degradation and urinary excretion of catabolites by humans. J Agric Food Chem 58: 1296–1304. 20041649 10.1021/jf9032975

[38] Willaimson G, Clifford MN (2010) Colonic metabolites of berry polyphenols: the missing link to biological activity? Br J Nutr 104 (Suppl 3)S48–S66. 20955650 10.1017/S0007114510003946

[39] Sánchez-Patán F, Monagas M, Moreno-Arribas MV, Bartolomé B (2011) Determination of microbial phenolic acids in human faeces by UPLC-ESI-TQ MS. J Agric Food Chem 59: 2241–2247. 21366314 10.1021/jf104574z

[40] Calani L, Del Rio D, Luisa Callegari M, Morelli L, Brighenti F (2012) Updated bioavailability and 48 h excretion profile of flavan-3-ols from green tea in humans. Int J Food Sci Nutr 63: 513–521. 22133145 10.3109/09637486.2011.640311

[41] Mulder TP, Rietveld AG, van Amelsvoort JM (2005) Consumption of both black tea and green tea results in an increase in the excretion of hippuric acid into urine. Am J Clin Nutr 81(1 Suppl): 256S–260S. 10.1093/ajcn/81.1.256S 15640488

[42] Larrosa M, Luceri C, Vivoli E, Pagliuca C, Lodovici M, et al (2009) Polyphenol metabolites from colonic microbiota exert anti-inflammatory activity on different inflammation models. Mol Nutr Food Res 53: 1044–1054. 19557820 10.1002/mnfr.200800446

[43] Zheng LT, Ryu GM, Kwon BM, Lee WH, Suk K (2008) Anti-inflammatory effects of catechols in lipopolysaccharide-stimulated microglia cells: inhibition of microglial neurotoxicity. Eur J Pharmacol 588: 106–113. 18499097 10.1016/j.ejphar.2008.04.035

[44] Karlsen A, Retterstøl L, Laake P, Paur I, Bøhn SK, et al (2007) Anthocyanins inhibit nuclear factor-kappaB activation in monocytes and reduce plasma concentrations of pro-inflammatory mediators in healthy adults. J Nutr 137: 1951–1954. 17634269 10.1093/jn/137.8.1951

[45] Tomé-Carneiro J, Gonzálvez M, Larrosa M, Yáñez-Gascón MJ, García-Almagro FJ, et al.. (2012) Grape resveratrol increases serum adiponectin and downregulates inflammatory genes in peripheral blood mononuclear cells: a triple-blind, placebo-controlled, one-year clinical trial in patients with stable coronary artery disease. Cardiovasc Drugs Ther [Epub ahead of print] 10.1007/s10557-012-6427-8 PMC3555235 23224687

[46] Selma MV, Espín JC, Tomás-Barberán FA (2009) Interaction between phenolics and gut microbiota: role in human health. J Agric Food Chem 57: 6485–6501. 19580283 10.1021/jf902107d

[47] Jaganath IB, Mullen W, Lean ME, Edwards CA, Crozier A (2009) In vitro catabolism of rutin by human fecal bacteria and the antioxidant capacity of its catabolites. Free Radic Biol Med 47: 1180–1189. 19647790 10.1016/j.freeradbiomed.2009.07.031

[48] Lamprecht M, Frauwallner A (2012) Exercise, intestinal barrier dysfunction and probiotic supplementation. Med Sport Sci 59: 47–56. 23075554 10.1159/000342169

[49] Chun OK, Floegel A, Chung SJ, Chung CE, Song WO, et al (2010) Estimation of antioxidant intakes from diet and supplements in U.S. adults. J Nutr 140: 317–324. 20032488 10.3945/jn.109.114413

[50] Hursel R, Viechtbauer W, Dulloo AG, Tremblay A, Tappy L, et al (2011) The effects of catechin rich teas and caffeine on energy expenditure and fat oxidation: a meta-analysis. Obes Rev 12: e573–e581. 21366839 10.1111/j.1467-789X.2011.00862.x

