# Supplementary figures and images for: Influence of a Polyphenol-Enriched Protein Powder on Exercise-Induced Inflammation and Oxidative Stress in Athletes: A Randomized Trial Using a Metabolomics Approach
Source: PLoS One. 2013 Aug 15;8(8):e72215. doi: 10.1371/journal.pone.0072215 (PMC3744465; doi:10.1371/journal.pone.0072215)

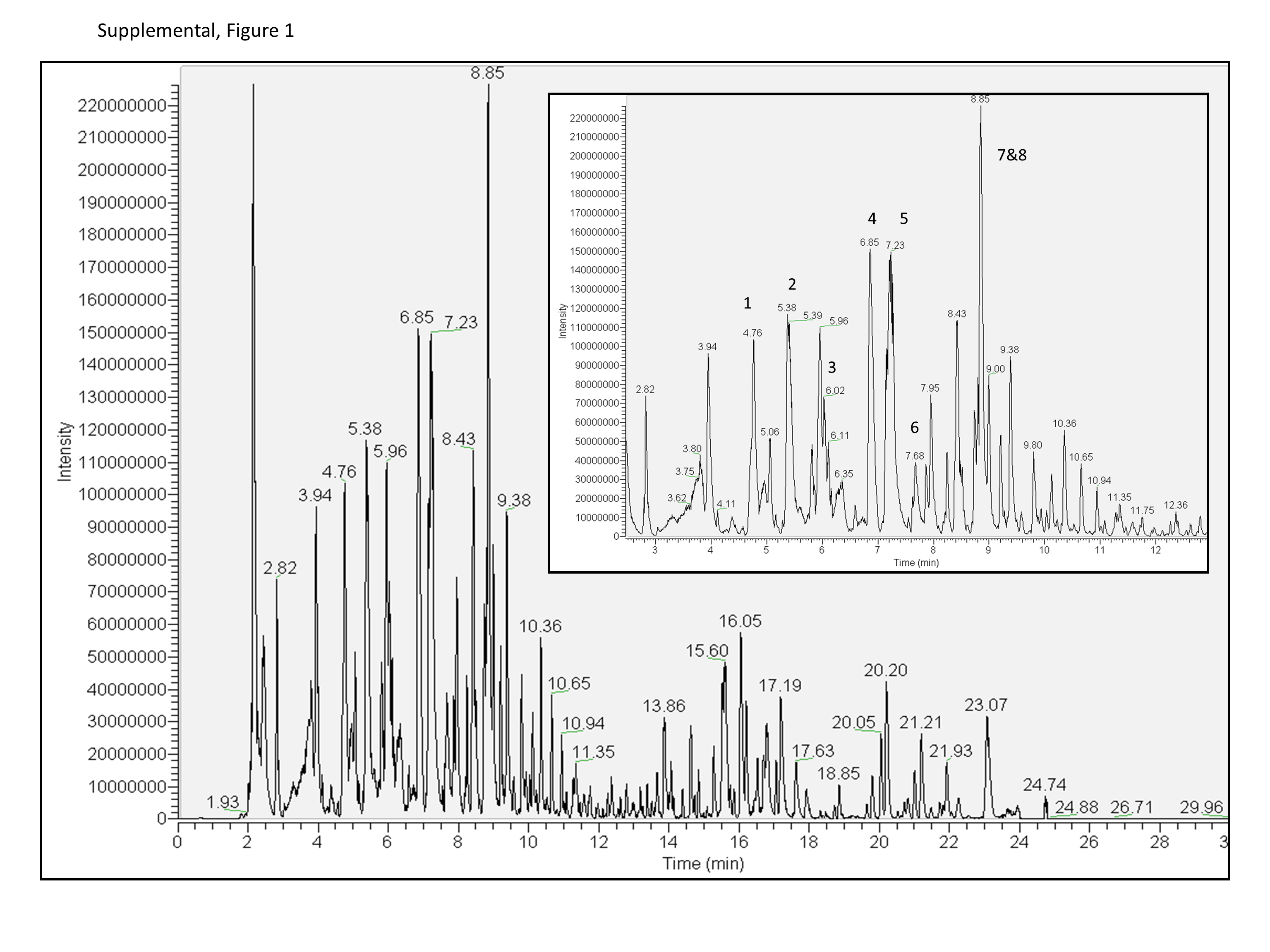

Supplement: Figure S1 — Negative ion mode total ion chromatogram (TIC) of green tea SPI (top), insert is expanded region (2–12 min). Identified flavan-3-ols are: gallocatechin (1), epigallocatechin (2), catechin (3), epicatechin (4), epigallocatechin gallate (5), gallocatechin gallate (6), catechin gallate (7) and epicatechin gallate (8). (TIF) [file pone.0072215.s001.tif]

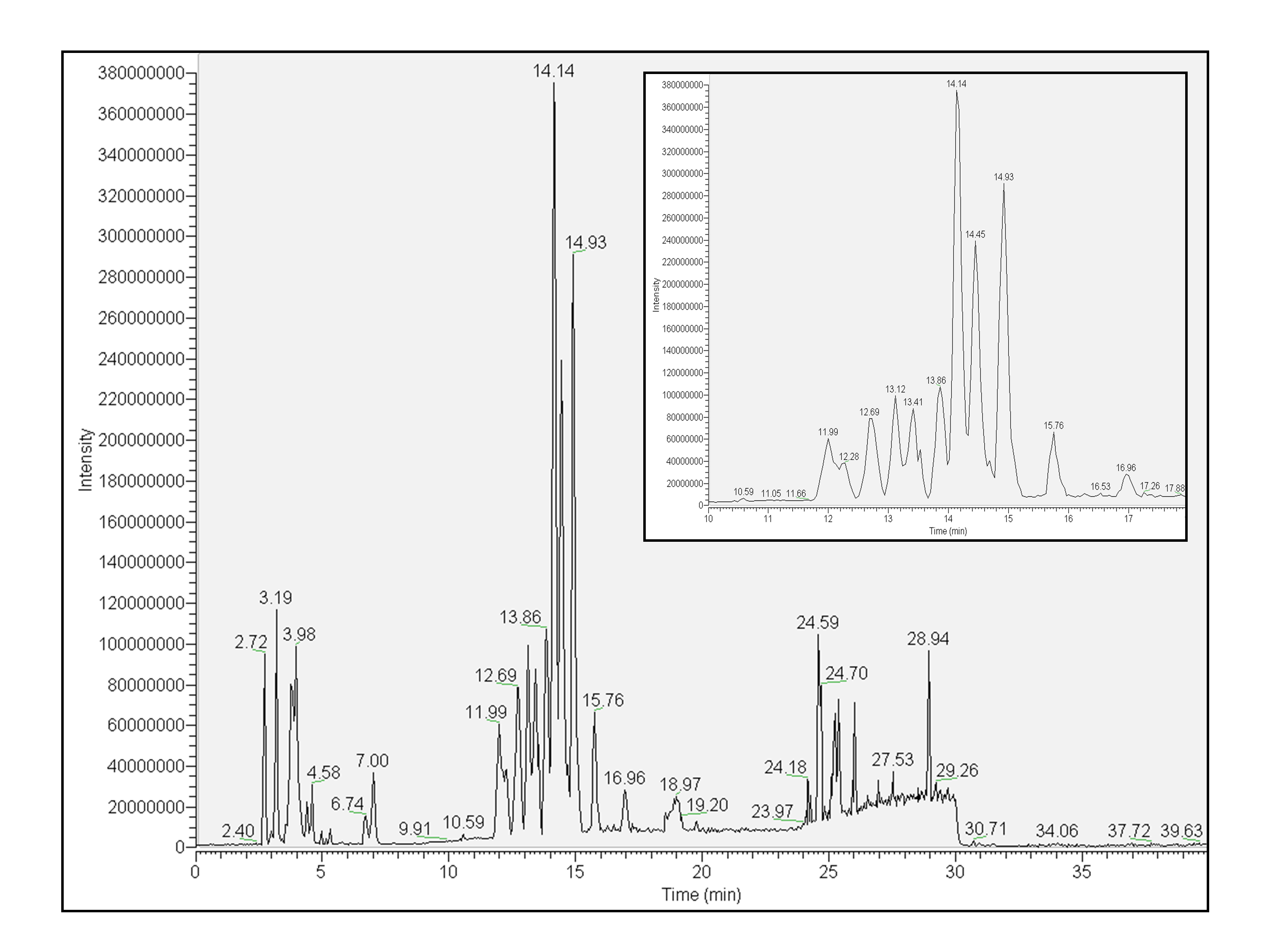

Supplement: Figure S2 — Positive ion mode total ion chromatogram (TIC) of bluebery SPI (top), insert is expanded region (10–18 min). Identified compounds by rentention time are: 11.99 - delphinidin 3-galactoside (Del-gal); 12.28 - delphinidin 3-glucoside (Del-glu); 12.69 - delphinidin pentose conjugate (Del-pent); 13.12 - petunidin 3-galactoside (Pet-gal); 13.41 - petunidin 3-glucoside (Pet-glu); 13.86 - petunidin pentose conjugate (Pet-pent); 14.14 - malvidin 3-galactoside (Mal-gal); 14.45 - malvidin 3-glucoside (Mal-glu); 14.93 - malvidin pentose conjugate 1 (Mal-pent1); 15.76 - malvidin pentose conjugate 2 (Mal-pent2) and 16.96 - malvidin acetoyl hexose conjugate (Mal-AcHex). (TIF) [file pone.0072215.s002.tif]
