# Supplementary material for: Influence of a Polyphenol-Enriched Protein Powder on Exercise-Induced Inflammation and Oxidative Stress in Athletes: A Randomized Trial Using a Metabolomics Approach
Source: PLoS One. 2013 Aug 15;8(8):e72215. doi: 10.1371/journal.pone.0072215 (PMC3744465; doi:10.1371/journal.pone.0072215)
Supplement: Table S1 — Mass spectral data for polyphenolic compounds identified in acidified methanol extract of blueberry SPI. (DOCX) [file pone.0072215.s003.docx]

**Table S1:** Mass spectral data for polyphenolic compounds identified in acidified methanol extract of blueberry SPI.

| **RT,**  GC  **min** | **MS** | **MS^n^** | **Compound ID ^a^** |
| --- | --- | --- | --- |
| 11.99 | 465 | (465)^2^ 303 | delphinidin galactoside (assigned by RT compared to 12.28 min) |
| 12.28 | 465 | (465)^2^ 303 | delphinidin glucoside |
| 12.69 | 435 | (435)^2^ 303 | delphinidin + pentose (probably arabinoside – MAL work) |
| 12.78 | 449 | (449)^2^ 287 | cyanidin 3-galactoside **^b^** (assigned by RT compared to 13.08 min) |
| 13.08 | 449 | (449)^2^ 287 | cyanidin 3-glucoside **^b^** |
| 13.12 | 479 | (479)^2^ 317 | petunidin 3-galactoside (assigned by RT compared to 13.41 min) |
| 13.41 | 479 | (479)^2^ 317 | petunidin 3-glucoside |
| 13.51 | 419 | (419)^2^ 287 | cyanidin + pentose (probably arabinoside – MAL work) |
| 13.86 | 449 | (449)^2^ 317 | petunidin + pentose (probably arabinoside – MAL work) |
| 14.14 | 493 | (493)^2^ 331 | malvidin galactoside (assigned by RT compared to 14.45 min) |
| 14.45 | 493 | (493)^2^ 331 | malvidin glucoside |
| 14.93 | 463 | (463)^2^ 331 | malvidin + pentose (probably arabinoside – MAL work) |
| 15.69 | 355 | (355)^2^ 163; (163)^3^ 145, 135 | **Unknown ^c^** |
| 15.76 | 463 | (463)^2^ 331 | malvidin + pentose (maybe xyloside? since arabinoside appears at 14.93 min) |
| 16.96 | 535 | (535)^2^ 331 | malvidin + acetoyl + hexose (probably 6-acetyl glucoside/galactoside – MAL work) |

**^a^** Tentative identification based on [M+H]^+^, MS^2^ of aglycone and fragment mass of glycoside moiety by comparisons with Wu *et al J. Agric. Food Chem.* **2005**, 53, 2589-2599. **Red Bold** = unidentified compound; **^b^** Does not appear in TIC but appears in IEC for [M+H]^+^ = 287); **^c^** Does not appear in TIC but appears in IEC for [M+H]^+^ = 355).
