# Supplementary material for: Influence of a Polyphenol-Enriched Protein Powder on Exercise-Induced Inflammation and Oxidative Stress in Athletes: A Randomized Trial Using a Metabolomics Approach
Source: PLoS One. 2013 Aug 15;8(8):e72215. doi: 10.1371/journal.pone.0072215 (PMC3744465; doi:10.1371/journal.pone.0072215)
Supplement: Table S2 — Mass spectral data for polyphenolic compounds identified in acidified methanol extract of green tea SPI. (DOCX) [file pone.0072215.s004.docx]

**Table S2:** Mass spectral data for polyphenolic compounds identified in acidified methanol extract of green tea SPI.

| **RT,**  GC  **min** | **MS** | **MS^n^** | **Compound ID ^a^** |
| --- | --- | --- | --- |
| 2.82 | 191 | (191)^2^:111,173 | quinic acid |
| 3.94 | 343 | (343)^2^:191,169 | 5-galloylquinic acid |
| 4.76 | 305 | (305)^2^:179,219,221,261 | **gallocatechin** |
| 5.06 | 353 | (353)^2^:191,175,135 | 3-caffeoylquinic acid |
| 5.38 | 305 | (305)^2^:179,219,221,261 | **epigallocatechin** |
| 5.81 | 337 | (337)^2^:163 | **Unknown** |
| 5.96 | 353 | (353)^2^:173,171,191 | 5-caffeoylquinic acid |
| 6.02 | 289 | (289)^2^:245,205,179 | **catechin** |
| 6.85 | 289 | (289)^2^:245,205,179 | **epicatechin** |
| 7.23 | 457 | (457)^2^:169,331,305,287 | **epigallocatechin gallate** |
| 7.68 | 457 | (457)^2^:169,331,305,287 | **gallocatechin gallate** |
| 7.95 | 479 | (479)^2^:316,317 | myricetin + hexose |
| 8.25 | 755 | (755)^2^:609,447,301 | quercetin + rhamnose + hexose |
| 8.43 | 609 | (609)^2^:301 | quercetin-3-O-rutinoside |
| 8.74 | 463 | (463)^2^:301 | quercetin + hexose |
| 8.85 | 441 | (441)^2^:289,169,271,331 | **catechin gallate** |
| 8.92 | 441 | (441)^2^:289,169,271,331 | **epicatechin gallate** |
| 9.00 | 593 | (593)^2^:285 | kaempferol + rhamnose + hexose |
| 9.21 | 447 | (447)^2^:284,285 | kaempferol galactoside |
| 9.38 | 447 | (447)^2^:285 | kaempferol glucoside |
| 9.80 | 425 | (425)^2^:273,255,169 | **Unknown** |
| 10.36 | 901 | (901)^2^:755,609,447,301 | quercetin + rhamnose + hexose + rhamnose + rhamnose |
| 10.65 | 885,755,577,301 | (885)^2^:739,431,285  (755)^2^:609,447,301 | kaempferol + rhamnose + rhamnose + hexose + rhamnose  quercetin + rhamnose + hexose + rhamnose |

**^a^ Black Bold** = definitive identification based on comparison with authentic standard; Regular Type = tentative identification based on mass spectral data and comparison with Del Rio *et al J. Agric. Food Chem.* **2004**, 52, 2807-2815; **Red Bold** = unidentified compound
