# Supplementary material for: Influence of a Polyphenol-Enriched Protein Powder on Exercise-Induced Inflammation and Oxidative Stress in Athletes: A Randomized Trial Using a Metabolomics Approach
Source: PLoS One. 2013 Aug 15;8(8):e72215. doi: 10.1371/journal.pone.0072215 (PMC3744465; doi:10.1371/journal.pone.0072215)
Supplement: Protocol S1 — Human research protection application and protocol summary. (DOC) [file pone.0072215.s006.doc]

**Protocol S1**

**Request for Review of Human Participant Research**

Appalachian Human Research Protection Program

IRB # *(To be filled out by IRB Administration)*

*Instructions:* ***Complete and send the request form electronically to*** [***irb@appstate.edu***](mailto:irb@appstate.edu)*.*

***Note:*** *checkboxes can be checked by putting an “x” in the box.*

**Section I: Study Description**

Study Title:

**Influence of Nutrasorb on performance and exercise-induced inflammation, oxidative stress, and changes in immune function in athletes: a metabolomics-based approach.**

Study Description: *Please describe briefly the objectives of the study with the purpose, research question and any relevant background information.*

**PROTOCOL SUMMARY**

Nutrasorb is a newly developed food product (see [www.nutrasorb.com](http://www.nutrasorb.com/)). This study will use the Nutrasorb soy protein product that is matrixed with polyphenols from blueberries, grapes, and green tea, and test for efficacy as a nutritional countermeasure to exercise-induced physiologic stress (i.e., immune dysfunction, inflammation, and oxidative stress).

**Sponsor**: Dole Foods.

**Study Design:**

Allocation:  Randomized
Endpoint Classification:  Inflammatory, oxidative stress, immune, and metabolomics biomarkers
Intervention Model:  Two treatments (Nutrasorb vs. placebo).
Masking:  Double-blinded, placebo controlled, randomized

Sample Size: N=38 (including 4 alternates)

Primary Purpose:  Assessment of the effect of Nutrasorb (soy protein with polyphenols) as a countermeasure to exercise-induced exercise inflammation, oxidative stress, and immune changes

Study Duration (per subject): 18 days.

Blood sample schedule: One blood draw at the following **four** time points: baseline, post-two weeks supplementation, post-three days endurance exercise, 14-h post endurance exercise

Each blood sample = 35 ml

Subject conditions: Subjects will consume 2 tablespoons Nutrasorb or placebo powder mixed in plain yogurt for 14 days, and during the 3-day period of intensified exercise bouts.

**Primary Outcome Measures (total of 136 blood samples):**

- Inflammatory cytokines: plasma IL-6, IL-8, IL-10, TNF-alpha, G-CSF, MCP-1
- Innate Immune Function: granulocyte and monocyte phagocytosis/oxidative burst activity
- Oxidative Stress and Capacity: RBC glutathione, protein carbonyls, plasma ORAC, FRAP, and F2-isoprostanes
- Glucose, CRP, Diagnostic Chemistries
- Metabolomics

**Study Treatment Cohort:**

| **Arms** | **Assigned Interventions** |
| --- | --- |
| Placebo | Soy protein powder WITHOUT polyphenols |
| Nutrasorb | Soy protein isolate matrixed with polyphenols from blueberries, green tea extract, and Muscadine grapes |

1. Principal Investigator(s) and responsible faculty member if student is the PI: David C. Nieman

Department(s): HLES (Human Performance Lab at the North Carolina Research Campus)

1. By submitting this request, the Principal Investigator (and responsible faculty member if PI is a student) accepts responsibility for ensuring that all members of the research team: 1) complete the required CITI training and any other necessary training to fulfill their study responsibilities, 2) follow the study procedures as described in the IRB approved application and comply with *Appalachian’s Guidelines for the Review of Research Involving Human Subjects* and all IRB communication and 3) uphold the rights and welfare of all study participants.

The parties (i.e., the IRB and the Principal Investigator and responsible faculty member if PI is a student) have agreed to conduct this application process by electronic means, and this application is signed electronically by the Principal Investigator and by the responsible faculty member if a student is the PI.

My name and email address together constitute the symbol and/or process I have adopted with the intent to sign this application, and my name and email address, set out below, thus constitute my electronic signature to this application.

David C. Nieman niemandc@appstate.edu

PI Name PI Email address

__     ___

Responsible Faculty Name if PI is a student Responsible Faculty Email address if PI is a student

| 1. Do you plan to publish or present off-campus? |  | No | x | Yes |
| --- | --- | --- | --- | --- |
| 1. Does this research involve any out-of-country travel? | x | No |  | Yes |

| **7**. Type of Research, check all that apply: | | | x | Faculty Research |  | Dissertation/Thesis/Honor’s Thesis |
| --- | --- | --- | --- | --- | --- | --- |
|  |  | Product of Learning |  | Class Project – Course Number: | | |
|  |  | Educational Research Involving Normal Education Practices | | | | |
|  |  | Other: describe | | | | |

| **8**. Source of Funding |  | Not Funded |  | Funds Awarded | x | Funds Pending |
| --- | --- | --- | --- | --- | --- | --- |
|  |  | Federally Funded |  | University Funded: describe | | |

If funds awarded/pending, provide sponsor name, Sponsored Programs number:

**Dole Foods Incorporated**

*Attach a copy of the contract/grant/agreement.*

**9.** Is another institution engaged in the research (i.e., an agent of another institution will obtain informed consent, interact with participants to obtain information, or access private identifiable information about participants)?

|  | x | No |  | Yes | If yes, list institution(s) and whether that IRB will review or rely on the ASU IRB |
| --- | --- | --- | --- | --- | --- |

**10.** What, if any, relationship exists between the researcher(s) and agencies (e.g., schools, hospitals, homes) involved in the research? *Attach statement of approval (e.g., letter of agreement) from any agencies that will be involved with the research.*

**Section II: Research Personnel**

Enter each team member (including PI) in the table below. (*A member of the research team is defined as one who will: 1) access participants’ private identifiable information, 2) obtain informed consent* ***or*** *3)* *interact with participants.)*

| **Name** | **Role** (e.g., PI, co-I, Research Assistant, Research Coord., Faculty Advisor, etc.) | **Responsibilities**: Select all that apply from the list of Responsibilities below  (e.g., “a, b, c”) | **Receive IRB Correspondence** (Y/N)?  If yes, provide preferred email address. |
| --- | --- | --- | --- |
| David C. Nieman | PI | A,b,c,d,e,f,g,l,j,m | knabam@appstate.edu |
| Amy M. Knab | Co-I | A,b,c,d,e,f,g,I,j,m | niemandc@appstate.edu |
| Lynn Cialdella Kam | Co-I | A,b,c,d,e,f,g,j,m | kamla@appstate.edu |
| Andrew Shanely | Co-I | A,b,c,d,e,f,g,I,j,m | [shanelyra@appstate.edu](mailto:shanelyra@appstate.edu) |
| Dru Henson | Co-I | A,b,c,d,e,f,g,I,j,m | hensonda@appstate.edu |
| Mary Pat Meaney | Co-I | A,b,c,d,e,f,g,j,m | meaneymp@appstate.edu |
| Pamela Lambeth | Research Assistant | A,b,c,d,e,f,g,j,m | lambethpg@appstate.edu |
| Dustin Dew | Research Assistant | A,b,c,d,e,f,g,j,m | dd73434@appstate.edu |
| Eric Vail | Research Assistant | C,g,I,j | appleslotsaapples@yahoo.com |

**(Note:** If you need additional room, you can add rows by going to right click, insert, and then insert rows below. Personnel changes made after IRB approval can be submitted via email with the above information.)

**Responsibilities:**

| **a.** Screens potential participants | **h**. Conducts physical exams |
| --- | --- |
| **b.** Obtains Informed Consent | **i**. Collects biological specimens (e.g., blood samples) |
| **c.** Has access to identifiable data | **j**. Conducts study procedures |
| **d.** Administers survey | **k**. Dispenses medications |
| **e**. Conducts interviews | **l.** Supervises exercise |
| **f.** Enters subject data into research records | **m.** Educates participants, families, or staff |
| **g**. Analyzes data with identifiable information | **n.** Other: describe |

**Note**: In some cases, expertise to perform study procedures (e.g., blood draws, interviewing participants about sensitive topics) should be documented by the IRB to show that risks to participants is minimized. The IRB uses the Research Personnel Form to document investigator expertise.

**Section III: Conflict of Interest**

**1.** Are there any known or potential conflicts of interest related to this research?

*Conflict of interest relates to situations in which financial or other personal considerations may compromise or involve the potential/have the appearance for compromising an employee’s objectivity in meeting University responsibilities including research activities.*

*Examples of conflicts of interest include but are not limited to: an investigator has equity in a business that conducts research in a related area; an investigator will receive an incentive/bonus based on the number or speed of enrollment or outcome of a study; or an investigator or family member is a consultant, holds an executive position or serves as a board member of the research sponsor or its holdings.*

If yes, describe and explain how participants will be protected from the influence of competing interests.

| x | No |  | Yes |
| --- | --- | --- | --- |

**Section IV: Participant Population and Recruitment**

**1.** Number of participants sought: 38

**2.** Targeted Participant Population (check all that apply):

| x | Adults (>= 18 yrs old) |  | College Students (only 18 or older) |
| --- | --- | --- | --- |
|  | Minors (< 18 yrs old) Age range: |  | College Students (under 18 may participate) |
|  | Minorities |  | Prisoners |
|  | Institutionalized Participants |  | Cognitively or emotionally impaired |
|  | Inpatient participants |  | Non-English speaking |
|  | Outpatient participants |  | Pregnant Participants |
|  | International research |  | Employees of a profit or non-profit organization |

**3**. Federal regulations have established guidelines for the equitable selection of participants. Are participants an appropriate group to bear the burdens of this research?

| x | Yes |  | No If no, please explain: |
| --- | --- | --- | --- |

Are participants a subset of the population most likely to receive the benefits of this research?

| x | Yes |  | No If no, please explain: |
| --- | --- | --- | --- |

**4.** Explain any inclusion and exclusion criteria for the study:

**SUBJECTS:**

Subjects will include 38 male or female endurance athletes (ages 18-55) who are competitive runners or cyclists and are capable of exercising for 2.5-h at a high intensity (70-75% VO2_max_) on a treadmill or CompuTrainer in a laboratory setting. Runners must regularly compete in marathon type events, and cyclists must be competitive Category 1-5. Subjects must agree to train normally, stay weight stable, and avoid the use of large-dose vitamin/mineral supplements (above 100% of recommended dietary allowances), herbs, and medications known to affect inflammation and immune function during the project. Exclusion criteria: Regularly take supplements, or other medicines known to effect inflammation. Subjects must be rated as “low risk” for disease, and have none of the items listed under the medical history, symptoms, or other health issues section of the screening questionnaire (ACSM).

| **5.** Recruitment Procedures (how will you find participants?) | | | |
| --- | --- | --- | --- |
|  |  | | Student Subject Pool; indicate pool: |
|  | x | Email/Mailing/Handout | |
|  | x | Website ad/Newspaper ads/Flyers/Postings | |
|  |  | School children with request sent to parents | |
|  | x | Participants will be approached by staff members | |
|  |  | Other (explained below) | |

*A copy of any recruitment materials must be submitted with this application.*

**6.** Explain details of recruitment (e.g., obtain list of student emails from Registrar’s office and send them recruitment email): Website ([www.ncrc.appstate.edu](http://www.ncrc.appstate.edu/)); advertising in the Kannapolis, NC area (newspaper articles, websites).

**7**. Does the research include any compensation, monetary inducements, or reimbursement for participation in this research study?

|  | No | x | Yes If yes, explain payment schedule: $400 to each subject completing the study (with proportional payment for percent of study completed) |
| --- | --- | --- | --- |

**Section V: Informed Consent Process**

**1.** Explain how informed consent will be obtained. *If applicable, include information about: the setting, whether participants will have an opportunity to ask questions, and the roles of any non-research personnel involved. If potential participants or their legally authorized representatives (e.g., parents) are non-English speaking, please explain how the investigator will identify these participants and ensure their ability to understand information about the study to provide consent.*

Voluntary informed consent; obtained in the NCRC Human Performance Laboratory operated by ASU; subjects can ask questions from study personnel administering the consent form.

**2.** If applicable, describe the safeguards in place to protect the rights and welfare of any vulnerable participants *(e.g., children, prisoners, pregnant persons, or any population that may be relatively or absolutely incapable of protecting their interests through the informed consent process).*

| **3.** Select factors that might interfere with informed consent: | | |
| --- | --- | --- |
|  | x | None known |
|  |  | Research will involve current students in a course/program taught by member of research team |
|  |  | Participants are employees whose supervisor is recruiting/requiring participation |
|  |  | Participants have a close relationship to research team |
|  |  | Other (please specify/indicate any relationship that exists between research team and participants): |
|  |  |

For selected factors, describe any efforts to mitigate:

**4**. Will participants sign a consent form?

| x | Yes |  | No |
| --- | --- | --- | --- |

If no, participants must still be provided with a statement regarding the research and one of the following criteria must be met and selected and followed:

The only record linking the participant and the research is the consent document and the principal risk is potential harm resulting from a breach of confidentiality, and the research is not FDA-regulated. Each participant will be asked whether he/she wants documentation linking the participant with the research and the participants wishes will govern; OR

|  |
| --- |

The research presents no more than minimal risk of harm and involves no procedures for which written consent is normally required outside of the research context.

|  |
| --- |

**5.** Are you requesting a modification to the required elements for informed consent for participants or legally authorized representatives?

| x | No |  | Yes If yes, address [criteria to waive elements of consent](http://www.orsp.appstate.edu/protections/irb/faqs#consent_waiver): |
| --- | --- | --- | --- |

**Section VI: Study Procedures**

**1.** Projected data collection dates: January 2012 to December 2012

**2.**  Describe research procedures as they relate to the use of human participants. *Information should include* *what participants will be asked to do, duration of procedures, and frequency of procedures*.

***Research Design:***

Phytochemicals are chemicals produced by plants, and include tannins, lignins, and flavonoids. The largest and best studied polyphenols are the flavonoids, with more than 6,000 identified and classified into at least six subgroups: flavonols, flavones, flavanones, flavanols (and their oligomers, proanthocyanidins), anthocyanidins, and isoflavonoids. Flavonoids are widely distributed in plants and function as plant pigments, signaling molecules, and defenders against infection and injury.

The research focus of the ASU Human Performance Laboratory (NCRC) is to test various nutritional agents for their capacity to attenuate oxidative stress, inflammation, muscle soreness, and immune changes following intensive exercise, and thus lower the magnitude of physiologic stress and risk of upper respiratory tract infection. Our data support findings from other research teams that flavonoid-rich plant extracts and unique flavonoid-nutrient mixtures used as daily supplements to the normal diet for at least two weeks help counter exercise-induced oxidative stress, inflammation, muscle soreness, and immune perturbations.

In this research project, 38 endurance athletes (male and female runners and cyclists) will complete baseline testing and then be randomized to either Nutrasorb or placebo groups. Two athletes in each group will be designated as reserve subjects to replace subjects who drop out of the study. After two weeks of supplementation, subjects (N=34) will complete three consecutive days of intense bouts of 2.5 hours exercise (running on treadmill or cycling on CompuTrainers) at 70-75% VO_2max_ with a 15 minute time trial at the end (with total distance measured). Thus, subjects will complete 2.25 hours of exercise and then participate in a 15 minute time trial (for a total of 2.5 hours exercise). The 15-minute time trial will serve as a performance measure and magnify inflammation, oxidative stress, and immune dysfunction (as determined in prior studies from our laboratory). Subjects will complete this protocol three days in a row. During the exercise bouts, all subjects will receive water ad libitum; but the subjects in the treatment group will receive one dose of Nutrasorb or placebo (i.e., one tablespoon of the powder) one hour into the 2.5-h exercise bout.


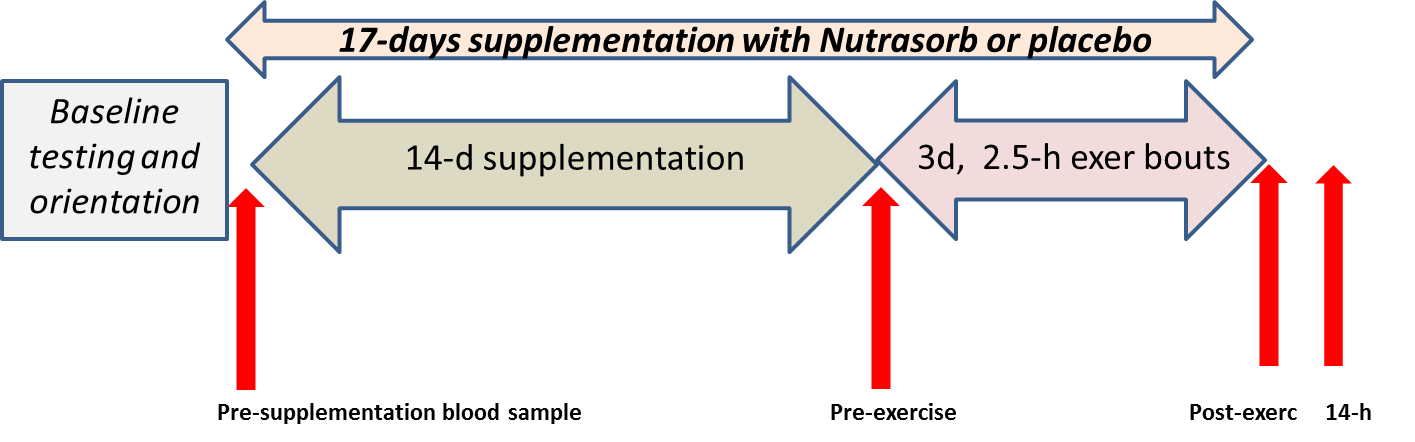


**Research Methods:**

- 1. **BASELINE TESTING**: Two weeks prior to the 3-day period of exercise (2.5-h exercise bouts), subjects will report to the NCRC Human Performance Lab for an appointment sometime between 2:30 and 5:30 pm for orientation and fitness testing (about 60-75 minutes). Cyclists will be tested for maximal aerobic fitness (i.e. VO_2max_) on a Lode cycle ergometer with a metabolic cart. Subjects will start cycling at a workload of 150 Watts and then increase power by 25 Watts every two minutes until they can no longer cycle due to fatigue. Runners will start walking on the treadmill at a speed of 1.7 miles/hr and a grade of 10%. The speed and grade will increase every 3 minutes until they can no longer run due to fatigue. During graded maximal exercise testing, subjects will breathe through a facemask that allows the metabolic system to measure oxygen consumption, ventilation, and breathing rate at maximal exercise. Body composition (i.e., percent body fat) will be measured with the BodPod system, and subjects will sit inside a small pod dressed in a tight-fitting swimsuit or bicycle racing shorts. Subjects will be randomized to Nutrasorb or placebo groups, and given dates for their participation in the study, including the pre-study blood draw (blood draw #1) and the start of supplementation. For a 3-day period prior to the first blood draw, subjects will keep a 3-day food record for analysis using Food Processor.
  2. **NUTRASORB/PLACEBO SUPPLEMENTATION:** Supplementation will last for 17 days (14-day supplementation period and 3-day intensified exercise period), and involves one tablespoon of Nutrasorb or placebo powder mixed in 6 oz plain yogurt in the morning, and then again at lunch. Compliance to the supplementation regimen will be followed through weekly email messages. Nutrasorb ([www.nutrasorb.com](http://www.nutrasorb.com/)) is a newly developed product that uses soy protein isolate (and other products) to capture polyphenols from various fruits and vegetables. Nutrasorb is a healthy, nutritious, all-GRAS (Generally Recognized As Safe) food material, and contains polyphenols from plant juices, teas and extracts leaving behind water, sugars and oils. The Nutrasorb-enhanced matrix delivers much greater amounts of phytonutrients in one serving than the original plant food. This study will use soy protein isolate with extracts from blueberries, Muscadine grapes, and green tea. One teaspoon of Nutrasorb can hold approximately 300 mg of flavonoids (the most abundant of the polyphenols or plant molecules) and 4 grams of protein. This study will use a daily dose of 2 tablespoons Nutrasorb (i.e., six teaspoons) or approximately 1,800 mg flavonoids from blueberries, Muscadine grapes, and green tea, and 24 grams protein. The average American consumes 210 mg/day flavonoids from food. Thus this study will provide about nine times this level, similar to levels used in prior research conducted in our Human Performance Laboratory. The average American also consumes about 90 grams of protein per day and this study will add 24 grams protein. The placebo supplement will contain the soy protein isolate powder with food coloring, but without the fruit polyphenols. A post-study symptom log will be administered (see attached).
  3. **E XERCISE TRIALS:** Subjects will use the supplement for two weeks while exercising normally, and then participate in a 3-day period of intensified exercise (i.e., three 2.5-h exercise bouts). In the morning of the first exercise session, subjects will consume the normal supplement portion (one tablespoon of Nutrasorb or placebo in yogurt). A good breakfast with carbohydrate foods is recommended (and a food list will be provided). A standardized meal consisting of Boost Plus at 12 kcal/kg will be ingested at 12:00 noon. The normal lunch-time Nutrasorb or placebo supplement will be delayed until after the blood draw at 2:30 pm. Subjects will report to the Human Performance Laboratory at 2:30 pm and provide blood samples (Blood draw #2). Subjects will next ingest the Nutrasorb or placebo supplement dose (in water this time). At 3:00 pm, subjects will either run on a treadmill or cycle on their own bicycles on CompuTrainers for 2.25-h followed by a 15 min time trial at the fastest pace possible, with distance measured for a performance outcome. Water will be given ad libitum throughout the 2.5-h exercise bouts, with no other beverage or food allowed. Subjects will ingest another dose of the Nutrasorb or placebo supplement (in water) after one hour of exercise (around 4 pm). Heart rate and rating of perceived exertion (RPE) will be taken every 30 minutes during the bout, with oxygen consumption and ventilation measured after one hour of exercise. Subjects will repeat this schedule for the next two days, but without pre-exercise blood draws. Blood samples (Blood draw 3) will be taken immediately following the third exercise bout on the third day (5:30 pm). Blood samples (Blood draw 4) will be taken approximately 14-h post-exercise the following morning at 7:30 am. For the entire study, four 35-ml blood samples will be collected for each subject for a total of 140 ml. The symptom log will be administered during the final blood draw session.

**3**. Participants’ identification (check one):

Information is collected so that participants CANNOT be identified directly (by names, images or other identifiers) or indirectly (by linking responses to participants).

|  |
| --- |

Information is collected so that participants CAN be identified, either directly or indirectly, by the research team but identifying information will not be disclosed publicly.

| x |
| --- |

Information is collected so that participants CAN be identified, either directly or indirectly, by the research team and identifying information will be disclosed publicly.

|  |
| --- |

| **4.** Check all locations of study procedures that apply: | | |
| --- | --- | --- |
|  |  | N/A – online survey |
|  |  | Appalachian campus, indicate building: |
|  |  | School system(s): |
|  | x | Human Performance Lab, NCRC |
|  |  | Off-campus location(s). List: |

**5.** Data collection

| **5a.** Please check all data collection activities involved in this study: | |
| --- | --- |
| **x** | Paper Surveys / Questionnaires |
|  | Online Surveys / Questionnaires Name of Survey Provider: |
|  | Telephone Surveys / Questionnaires Name of Survey Provider: |
|  | Standardized Written / Oral / Visual Tests |
|  | Interviews |
|  | Focus Groups |
|  | Tasks |
|  | Public Observation |
|  | Classroom Observation/Work Site Observation |
|  | Voice, video, digital or image recordings made for research purposes |
|  | Materials (i.e., data, documents, records/specimens) that have been collected or will be collected for **non research** purposes |
|  |
|  | Collection or study of materials (i.e., data, documents, records/specimens) that are publicly available or if the information is recorded so that participants cannot be identified, directly or indirectly through identifiers |
|  |
|  | Materials (i.e., data, documents, records/specimens) that have been collected for another research project |
| **X** | Vigorous exercise and muscular strength testing |
| **x** | Other: Body fat percentage will be measured with the Bod Pod. |

**5b.** If your study does not involve biomedical procedures skip to question #6. Otherwise, select all data collection activities that apply:

| x | Blood samples by finger stick, heel stick, ear stick or venipuncture | | |
| --- | --- | --- | --- |
| Indicate the type of participants and how much blood will be drawn: | | | |
|  | | x | from healthy, non pregnant adults who weigh at least 110 pounds |
|  | |  | from other adults or children |
|  | | x | How many times per week will blood be drawn? **3/week at the most** |
|  | | x | How much blood will be drawn at one time? **35 ml** |
|  | | x | How much blood will be drawn in an 8-week period? **140 ml** |
|  | | x | How often will collection occur? **Once during baseline testing, then three times during one week period. Total of 4 blood draws.** |
| x | Noninvasive procedures to collect biological specimens for research purposes | | |
|  | Sterile Surgical/Invasive procedures | | |
| x | Banking of biological materials | | |
| x | Noninvasive procedures to collect data such as use of physical sensors applied to surface of body and electrocardiography (Bod Pod) | | |
|  |
|  | Procedures involving x-rays (e.g., DEXA scan for body composition) | | |
|  | Ingestion of wholesome foods without additives | | |
| x | Ingestion/application of substances other than wholesome foods without additives **(soy protein with extracted fruit polyphenols)** | | |
|  | Clinical study of a drug/medical device | | |
|  | Obtaining medical data from a health care provider, health plan or health care clearinghouse | | |
|  | Genetic Testing | | |
|  | Other: describe | | |

**5c.** Is this research FDA-regulated (i.e., It is an experiment that involves one or more of the following test articles: foods/dietary supplements that bear a nutrient content/health claim, infant formulas, food/color additives, drugs/medical devices/biological products for human use)?

| x | No |  | Yes |
| --- | --- | --- | --- |

**6.** Is deception involved?

|  | x | No |  | Yes If yes, please describe: |
| --- | --- | --- | --- | --- |

**7.** Does the data to be collected relate to any illegal activities (e.g., immigration status, drug use, abuse, assault)?

|  | x | No |  | Yes If yes, please describe: |
| --- | --- | --- | --- | --- |

**Section VII: Confidentiality and Safeguards**

**1**. In most cases, the research plan should include adequate provisions to protect the privacy of subjects. How will the confidentiality of participants be maintained (e.g., how will access to participants be controlled)?

Subjects will be assigned identification numbers, and all data entry and analysis will be conducted with statistical programs using ID numbers. Subject files will be stored in Pam Krasen’s or Dr. Nieman’s office under lock and key. Identifiable information will be deleted after two years. All blood samples will be coded with identification numbers only. These samples will be coded with identification numbers only. The link between the codes and identifying information will be destroyed after two years. Identifying information will be deleted before subject records are shared with Dole Foods.

1. Will collected data be monitored to ensure the safety of subjects (e.g., survey includes a question about suicidality so the investigator will…)?

|  |  | No | x | Yes If yes, please explain procedures to ensure safety of participants: No harmful use of the data is intended. Data written in reports will not be linked to individual subjects. Subjects will be protected by avoiding the use of personal names or photographs (unless consent is given). |
| --- | --- | --- | --- | --- |

**3**. Describe what will be done with the data and resulting analysis:

Research files will be kept in Nieman’s office for 5 years, and then shredded prior to removal. The data will be presented at research conferences and published in a research journal. Blood samples that remain after all analysis has been completed will be destroyed after two years. Identifiable information will be deleted after two years.

| **4.** Describe measures you are taking to safeguard study data (check all that apply): | | |
| --- | --- | --- |
|  |  | Data is not linked to identifying information |
|  | **x** | Maintain consent forms in a separate location from data |
|  | x | Using subject codes on all collected data and maintaining the key linking subject codes with  identifiable information in a separate location from data |
|  |  |
|  | x | Locking cabinets/doors. List location: Krasen’s and Nieman’s offices |
|  | x | Data kept in area with limited public access. List location: Krasen’s and Nieman’s offices |
|  | x | Password protected computers |
|  |  | Encryption |
|  | x | PDAs and removable media (e.g., CDs, etc.) will be kept in a secure location. List location: Nieman’s office |
|  |  | Other, please describe: |

**5.** Data Sharing

| **5a.** What type of data will be shared? (*Note: Sharing includes releasing, transmitting and providing access to outside of the research team.)* Check all that apply: | | |
| --- | --- | --- |
|  |  | Data collected anonymously |
|  |  | Anonymized or De-linked data. Identity was once associated with data/specimen but identifying information destroyed |
|  |  |
|  | **x** | Coded and linked data (Data is coded. With the code, the data may be linked back to identifiers, but the link back to identifiers will not be shared.) |
|  |  |
|  |  | Identifiable Data (e.g., names, email addresses, date of birth, IP addresses)  Indicate which secure method(s) of transmission will be used: |
|  |  |

| **5b.** If identifiable data will be shared within or outside of the research team, please explain how it will be shared (check all that apply): | | |
| --- | --- | --- |
|  |  | Secured Website. Please provide name of website: |
|  |  | Encrypted email |
|  |  | U.S. Postal Service or other trackable courier services |
|  |  | Fax in a secured area |
|  |  | Shared drive with password protection |
|  | x | Personal delivery by member of research team |
|  |  | Private telephone conversation to member of research team |
|  |  | Other, please describe: |

***6****. Secure Disposal: Note: consent forms should be stored for 3 years after study completion*.

| **6a.** How long will the data be stored? | | | | |
| --- | --- | --- | --- | --- |
|  |  | 1 year after study conclusion | x | 5 years after study conclusion |
|  |  | Indefinitely |  | Data without identifiers stored indefinitely |
|  |  | Other, please describe (e.g., sponsor requirements): | | |

| **6b.** How will data be destroyed? | | | | |
| --- | --- | --- | --- | --- |
|  | x | Paper will be shredded | x | Biological samples will be destroyed by: December 2014 |
|  | x | Destroy electronic files from computer/PDAs/removal media (CDs, diskettes) by: December 2014 | | |
|  |  | Other, please describe: | | |

**Section VIII: Risk and Benefits of Study**

**1.** The risks to participants must be reasonable in relation to anticipated benefits, if any, to participants and the importance of the knowledge that may be reasonably be expected to result. Select all applicable:

Participants of the study may directly benefit by (describe)*:*

|  |
| --- |

Society may benefit from the study by (describe): *Nutrasorb is a soy protein powder with extracted polyphenols from fruit, and may serve as dietary supplement for athletes during hard training to attenuate inflammation, muscle soreness, oxidative stress, and immune dysfunction.*

| x |
| --- |

**2.** Describe the potential risks (e.g., psychological, legal, physical, social harm, loss of confidentiality) to any individual participating in this project:

The study will involve four separate blood draws with 35ml of blood taken per draw (thus for the entire study 140ml). The following statement will be included in the consent form: “The risks of collecting a blood sample from you include the possibility of requiring more than one attempt to obtain the blood sample, local discomfort (pinch when the needle enters your skin), minor bruising or bleeding at the site (10%), or possible temporary lightheadedness, infection (<0.01%), or development of a blood clot (0.01%). The amount of blood being withdrawn is a little over two tablespoons during each visit and will not affect your ability to participate in normal daily activities, or the ability to perform the exercise endurance rides. A trained and experienced individual will perform the technique and your blood will be collected in a hygienic setting with sterile materials and biohazard protection measures to minimize these risks. In the rare case of exposure of your blood or tissue to research personnel, we will analyze your blood for HIV and hepatitis (a positive HIV or hepatitis test will be reported to you).”

**3.** Assessment of level of risk:

Risks (including physical, emotional, social, legal or financial) are the same as encountered in daily life or during the performance of routine physical or psychological examinations or tests (minimal risk).

|  |
| --- |

Risks are more than minimal in that either: a) the probability of harm or discomfort anticipated, or b) the magnitude of harm or discomfort anticipated is greater than that encountered in daily life.

| x |
| --- |

Information to be collected could cause participants to be at risk of criminal or civil liability if responses are disclosed outside of the research setting.

|  |
| --- |

Information to be collected could be damaging to participant’s financial standing, employability, or reputation if disclosed outside of the research setting.

|  |
| --- |

**4.** Describe procedures for protecting against, or minimizing, the potential risks: Blood will be drawn via venipuncture by a trained phlebotomist. Subjects will review a consent form that contains a statement about the potential risks associated with venipuncture. This study will only include cyclists and runners who are deemed at “low risk” using ACSM’s screening questionnaire (thus diminishing potential exercise-related incidents). Nutrasorb is made of natural products (soy protein and fruit polyphenols), and is a healthy, nutritious, all-GRAS (Generally Recognized As Safe) food material.

**5.** If human subject data/specimens will be used for future research that is not described above, please explain. (Future use of data/specimens should be disclosed to the participant in the informed consent.)

Please check any materials below that will be submitted with your application. Note: please submit as separate files.

| x | Recruitment wording |
| --- | --- |
| x | Consent form(s) |
|  | Letter(s) of Agreement |
|  | Research Personnel Form(s) |
| x | Instruments (Survey questions, interview questions, etc.) |
| x | Copy of grant/contract/agreement |
|  | Other (please describe): |

Please **send an electronic Word attachment (not scanned) of this application and any accompanying materials to** [**irb@appstate.edu**](mailto:irb@appstate.edu). Thank you for taking your time to promote ethical human participant research at Appalachian!


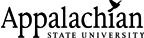


**Consent to Participate in Research**

**TITLE OF RESEARCH PROJECT: Influence of Nutrasorb on performance and exercise-induced inflammation, oxidative stress, and changes in immune function in athletes: a metabolomics-based approach.**

**Principal Investigator:** David C. Nieman, DrPH

**Department:** Human Performance Laboratory at the North Carolina Research Campus (NCRC), Appalachian State University (ASU); Department of Health, Leisure, and Exercise Science

**Contact Information:** David C. Nieman, 828-773-0056, [niemandc@appstate.edu](mailto:niemandc@appstate.edu); ASU Human Performance Lab at the NCRC, 600 Laureate Way, Kannapolis, NC 28081

**What is the purpose of this research?**

Intense and prolonged exercise (e.g., running a marathon race) can cause inflammation, muscle soreness, oxidative stress, and negative changes in the immune system. Our research team studies the effectiveness of novel food products and nutritional supplements in attenuating these indicators of physical stress. Nutrasorb is a newly developed food product (see [www.nutrasorb.com](http://www.nutrasorb.com/)). This study will use the Nutrasorb soy protein product that includes plant molecules (i.e., polyphenols) from blueberries, grapes, and green tea. We will test the effectiveness of 17-days supplementation with Nutrasorb in countering inflammation, oxidative stress, and negative immune changes caused by three days of intensive running or cycling 2.5 hours/day in a laboratory setting.

**Why am I being invited to take part in this research?**

You are invited to take part in this research if you are a healthy non-smoking male or female (age 18-55), and a trained competitive runner (i.e., have participated in half-marathons and full marathons) or cyclist (racing category 1-5 or professional), and can exercise for 2.5-h at a high intensity three days in a row. During the entire study, you will train normally, and avoid the use of large-dose vitamin/mineral supplements, herbs, and medications known to affect inflammation and immune function. Specifically, the use of any non-steroidal anti-inflammatory drugs (NSAIDS) such as ibuprofen is not permitted during the study.

**Are there reasons I should not take part in this research?**

I understand that I should **not** volunteer for this study if I am a smoker, under the age of 18 or over the age of 55 years, not healthy because of a chronic disease such as heart disease, cancer, stroke, arthritis, or diabetes, on any type of medication or nutritional supplement, unable to exercise for 2.5-hr at a vigorous intensity for three days in a row, and/or unwilling to adhere to all aspects of the study including Nutrasorb supplementation for 17 days.

**What will I be asked to do?**

The research procedures will be conducted at the Human Performance Laboratory (Room 1201, Plants for Human Health Institute Building, 600 Laureate Way), operated by Appalachian State University at the North Carolina Research Campus (NCRC) in Kannapolis, NC. You will come to the Human Performance Lab for baseline testing, a pre-supplementation blood draw, three days of 2.5-h exercise bouts, and the morning after the last exercise bout to provide a final blood sample. The total time involvement will be approximately 15 hours. Figure 1 summarizes the research design, and a detailed explanation follows.


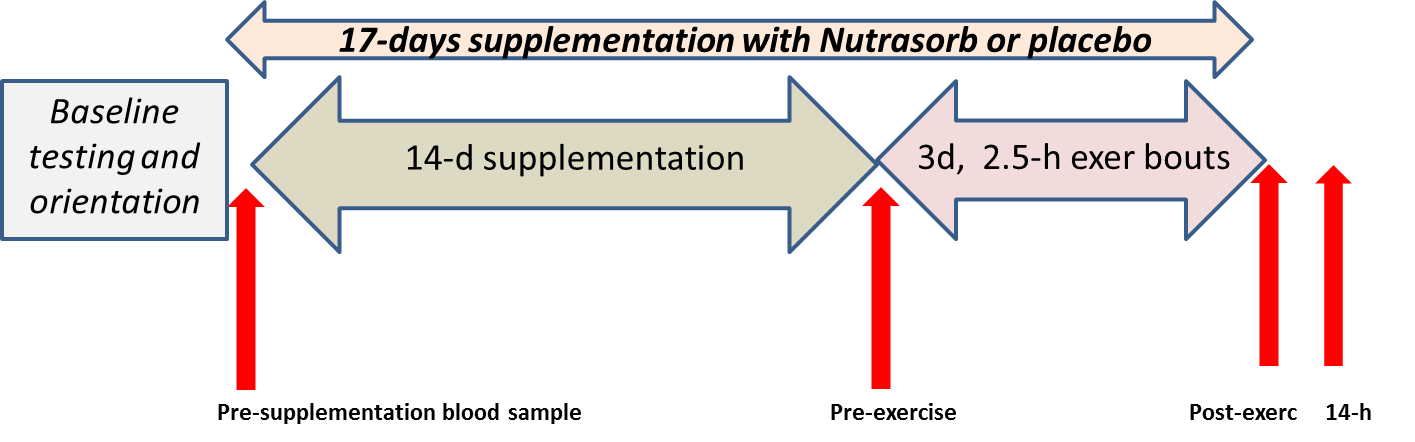


- 1. **BASELINE FITNESS TESTING:**  Two weeks prior to the 3-day period of exercise (2.5-h exercise bouts), you will report to the NCRC Human Performance Lab for an appointment sometime between 2:30 and 5:30 pm for orientation and fitness testing (about 60-75 minutes). If you are a cycler, your maximal aerobic fitness (i.e. VO_2max_) will be tested on a Lode cycle ergometer with a metabolic cart to measure oxygen consumption. You will start cycling at a workload of 150 Watts and then increase power by 25 Watts every two minutes until you can no longer cycle due to fatigue. If you are a runner, you will start walking on the treadmill at a speed of 1.7 miles/hr and a grade of 10%. The speed and grade will increase every 3 minutes until you can no longer run due to fatigue. During graded maximal exercise testing, you will breathe through a facemask that allows the metabolic system to measure oxygen consumption, ventilation, and breathing rate at maximal exercise. Body composition (i.e., percent body fat) will be measured with the BodPod system, and you will sit inside a small pod dressed in a tight-fitting swimsuit or bicycle racing shorts. You will be randomized to Nutrasorb or placebo groups, and given dates for participation in the study, including the pre-study blood draw and the start of supplementation.
  2. **NUTRASORB/PLACEBO SUPPLEMENTATION:** Supplementation will last for 17 days (14-day supplementation period and 3-day intensified exercise period), and involves one tablespoon of Nutrasorb or placebo powder mixed in 6 oz plain yogurt in the morning, and then again at lunch. (Plain yogurt will be supplied to you along with the Nutrasorb or placebo powder). Compliance to the supplementation regimen will be followed through weekly email messages. Nutrasorb ([www.nutrasorb.com](http://www.nutrasorb.com/)) is a newly developed product that uses soy protein isolate (and other products) to capture special plant molecules called polyphenols from various fruits and vegetables. Nutrasorb is a healthy, nutritious, safe food product, and contains polyphenols from plant juices, teas and extracts leaving behind water, sugars and oils. Nutrasorb delivers much greater amounts of polyphenols in one serving than the original plant food. This study will use soy protein isolate with extracts from blueberries, grapes, and green tea. One teaspoon of Nutrasorb can hold approximately 300 mg of flavonoids (the most abundant of the polyphenols or plant molecules) and 4 grams of protein. This study will use a daily dose of 2 tablespoons Nutrasorb (i.e., six teaspoons) or approximately 1,800 mg flavonoids from blueberries, grapes, and green tea, and 24 grams protein. The average American consumes 210 mg/day flavonoids from food. Thus this study will provide about nine times this level, similar to levels used safely in prior research conducted in our Human Performance Laboratory. The average American also consumes about 90 grams of protein per day and this study will add 24 grams protein. The placebo supplement will contain the soy protein isolate powder with food coloring, but without the fruit polyphenols. A post-study symptom log will be administered to obtain your responses to potential stomach and intestinal problems, muscle soreness, and mental alertness.
  3. **EXERCISE TRIALS:** You will use the supplement for two weeks while exercising and training normally, and then participate in a 3-day period of intensified exercise (i.e., three 2.5-h exercise bouts). In the morning of the first exercise session, you will consume the normal supplement portion (one tablespoon of Nutrasorb or placebo in 6 oz plain yogurt). A good breakfast with carbohydrate foods is recommended (and a food list will be provided). A standardized meal consisting of Boost Plus at 12 kcal/kg will be ingested at 12:00 noon (this will be provided to you). The normal lunch-time Nutrasorb or placebo supplement will be delayed until after the blood draw at 2:30 pm. You will report to the Human Performance Laboratory at 2:30 pm and provide a blood sample. You will next ingest the Nutrasorb or placebo supplement dose (one tablespoon powder in water). At 3:00 pm, you will either run on a treadmill or cycle on your own bicycle on CompuTrainers for 2.25-h followed by a 15 min time trial at the fastest pace possible, with distance measured for a performance outcome. You can consume water as desired throughout the 2.5-h exercise bouts, but no other beverage or food is allowed. You will ingest another dose of the Nutrasorb or placebo supplement after one hour of exercise (around 4 pm). Heart rate and rating of perceived exertion (RPE) will be taken every 30 minutes during the bout, with oxygen consumption and ventilation measured after one hour of exercise. You will repeat this schedule for the next two days, but without pre-exercise blood draws. A blood sample will be taken immediately following the third exercise bout on the third day (~5:30 pm). The fourth and final blood sample will be collected 14-h post-exercise the following morning at 7:30 am. For the entire study, four 35-ml blood samples will be collected for a total of 140 ml. The symptom log will be administered during the final blood draw session.

**What are possible harms or discomforts that I might experience during the research?**

To the best of our knowledge, the risk of harm for participating in this research study is no more than you (as a cyclist or runner) would experience in everyday life. Ingestion of Nutrasorb or the placebo powder is safe and has no known side effects. Nutrasorb ingredients are consistent with good nutrition and health.

The risks of collecting a blood sample (venipuncture) from you include the possibility of requiring more than one attempt to place the needle, local discomfort (pinch when the needle enters your skin), minor bruising or bleeding at the site (10%), possible temporary lightheadedness, infection (<0.01%), or development of a blood clot (<0.01%). The amount of blood being withdrawn during each of the four blood draw visits is about 35 ml (slightly more than two tablespoons) and will not influence your ability to participate in normal daily activities. The total amount of blood withdrawn for the entire study is 140 ml. A trained and experienced individual will perform the technique and your blood will be collected in a hygienic setting with sterile materials and biohazard protection measures to minimize these risks. In the rare case of research personnel exposure to your blood or tissue, we will analyze your blood for HIV and hepatitis (a positive HIV or hepatitis test will be reported to you).

The physical fitness tests and exercise bouts included in this study are safe and have no known risks for apparently healthy adults accustomed to exercise training. You will fill in a health screening questionnaire and must be classified as “low risk” to be included in this study. Several trained staff will supervise all physical fitness tests and exercise bouts. In the rare event of an injury during testing and exercise, standard emergency procedures in the Human Performance Laboratory will be followed. The ASU-NCRC Human Performance Lab is located within a few minutes of several agencies providing emergency treatment.

**What are possible benefits of this research?**

We do not know if you will get any benefits by taking part in this study. This research should help us learn more about whether ingesting Nutrasorb before and during exercise helps counter the inflammation, oxidative stress, and negative immune changes linked to heavy exercise. You will receive personal information about your aerobic fitness and body composition.

**Will I be paid for taking part in the research?**

We will pay you $400 for the time you volunteer while being in this study. If you do not complete the study, compensation will be pro-rated according to the percentage of study requirements completed. For example, if you complete the 2-week supplementation period and first day of exercise (2.5-h endurance bout), but then drop out of the study, you will receive $200. Current University policy requires the collection of Social Security numbers (or Appalachian Banner ID numbers) if study compensation is more than $20 for a single study or $599 for participation in multiple studies in a calendar year.  Since the compensation for this study is more than $20, you will need to provide your address and Social Security number (or Appalachian Banner ID number) when you complete the form for payment.

**How will you keep my private information confidential?**

Your information will be combined with information from other people taking part in the study. When we write up the study to share it with other researchers, we will write about the combined information. You will not be identified in any published or presented materials. To ensure that your information is kept confidential, identification numbers but not names will be used on all documents. All data entry and analysis will be conducted with statistical programs using coded identification. Your files will be stored in Dr. Nieman’s office under lock and key and identifiable information will be deleted after two years. All blood samples will be coded with identification numbers only, and samples that remain after all analysis in this study has been completed will be destroyed within two years of collection. With your permission, photos may be taken during the study and used in scientific presentations of the research findings. Your identity will not be revealed when the photos are presented.

**What if I get sick or hurt while participating in this research study?**

If you need emergency care while you are at the research site, it will be provided to you. If you believe you have been hurt or if you get sick because of something that is done during the study, you should call your doctor or if it is an emergency call 911 for help. In this case, tell the doctors, the hospital or emergency room staff that you are taking part in a research study and the name of the Principal Investigator. If possible, take a copy of this consent form with you when you go. Call the Principal Investigator (Dr. David Nieman, 828-773-0056) as soon as you can (he needs to know that you are hurt or ill). Costs associated with this care will be billed in the ordinary manner, to you or your insurance company. However, some insurance companies will not pay bills that are related to research costs. You should check with your insurance about this. Medical costs that result from research-related harm may also not qualify for payments through Medicare, or Medicaid. You should talk to the Principal Investigator about this, if you have concerns.

**Who can I contact if I have a question?**

The people conducting this study will be available to answer any questions concerning this research, now or in the future. You may contact the Principal Investigator at 828-773-0056. If you have questions about your rights as someone taking part in research, contact the Appalachian Institutional Review Board Administrator at 828-262-2130 (days), through email at irb@appstate.edu or at Appalachian State University, Office of Research and Sponsored Programs, IRB Administrator, Boone, NC 28608.

**Do I have to participate? What else should I know?**

Your participation in this research is completely voluntary. If you choose not to volunteer, there will be no penalty and you will not lose any benefits or rights you would normally have. If you decide to take part in the study you still have the right to decide at any time that you no longer want to continue. There will be no penalty and no loss of benefits or rights if you decide at any time to stop participating in the study.

This research project has been approved by the Institutional Review Board (IRB) at Appalachian State University.

This study was approved on:

This approval will expire on __________ unless the IRB renews the approval of this research.

**I have decided I want to take part in this research. What should I do now?**

The person obtaining informed consent will ask you to read the following and if you agree, you should sign this form:

- I have read (or had read to me) all of the above information.
- I have had an opportunity to ask questions about things in this research I did not understand and have received satisfactory answers.
- I understand that I can stop taking part in this study at any time.
- By signing this informed consent form, I am not giving up any of my rights.
- I have been given a copy of this consent document, and it is mine to keep.

Participant's Name (PRINT) Signature Date
